# Supplementary figures and images for: A comparison of transporter gene expression in three species of Peronospora plant pathogens during host infection
Source: PLoS One. 2023 Jun 1;18(6):e0285685. doi: 10.1371/journal.pone.0285685 (PMC10234565; doi:10.1371/journal.pone.0285685)

## Slide 1
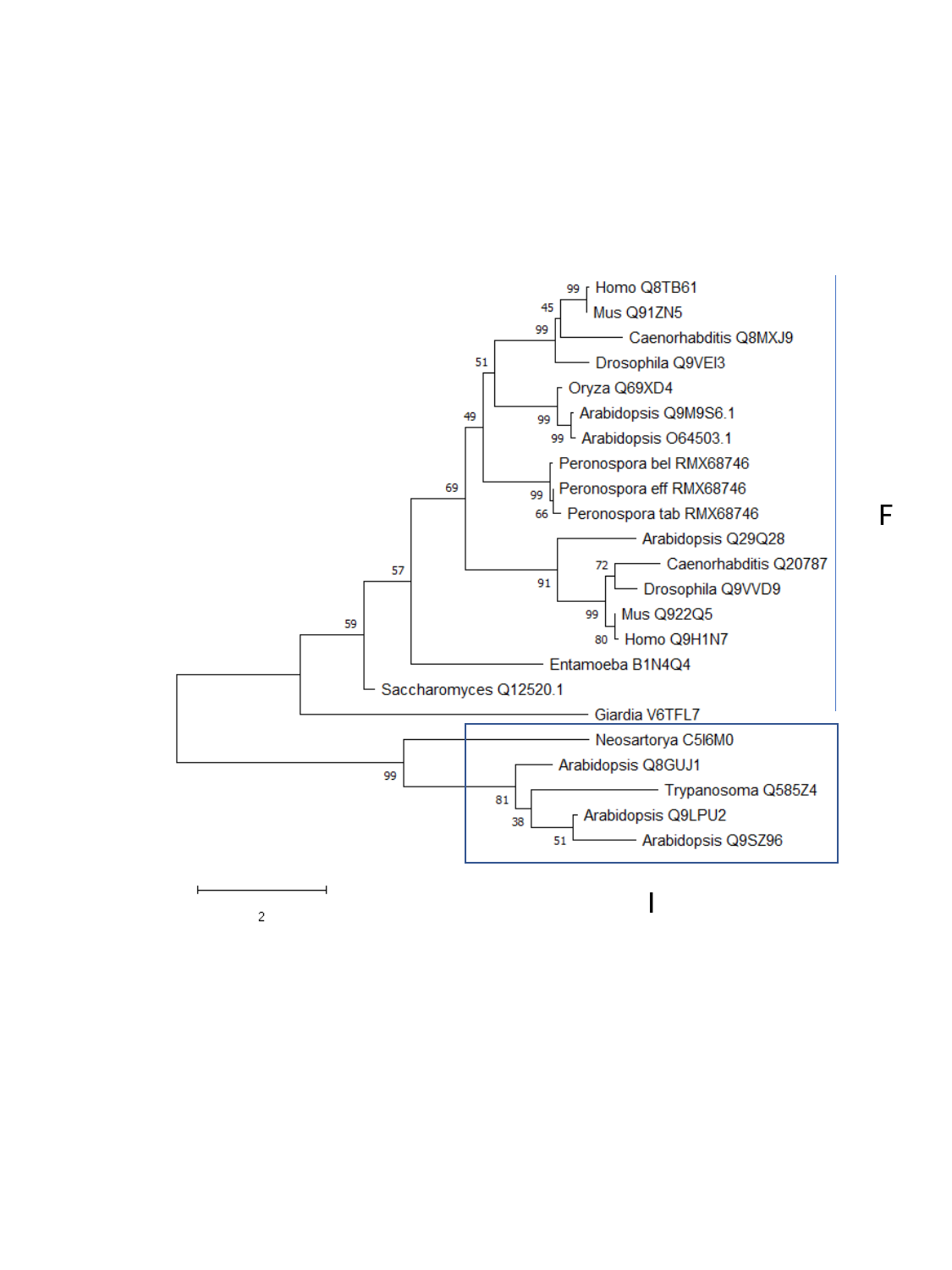

F
I

Supplement: S1 Fig — The neighbor-joining phylogenetic tree was constructed using the JTT model. The percentage of replicate trees in which the associated proteins clustered together in the bootstrap test (1000 replicates) are posted next to the branches. The F and I proteins, listed with their Uniprot accession numbers, formed two separate clades in the tree. The bar indicates the number of protein substitutions per site. (PPTX) [file pone.0285685.s001.pptx]

## Slide 1
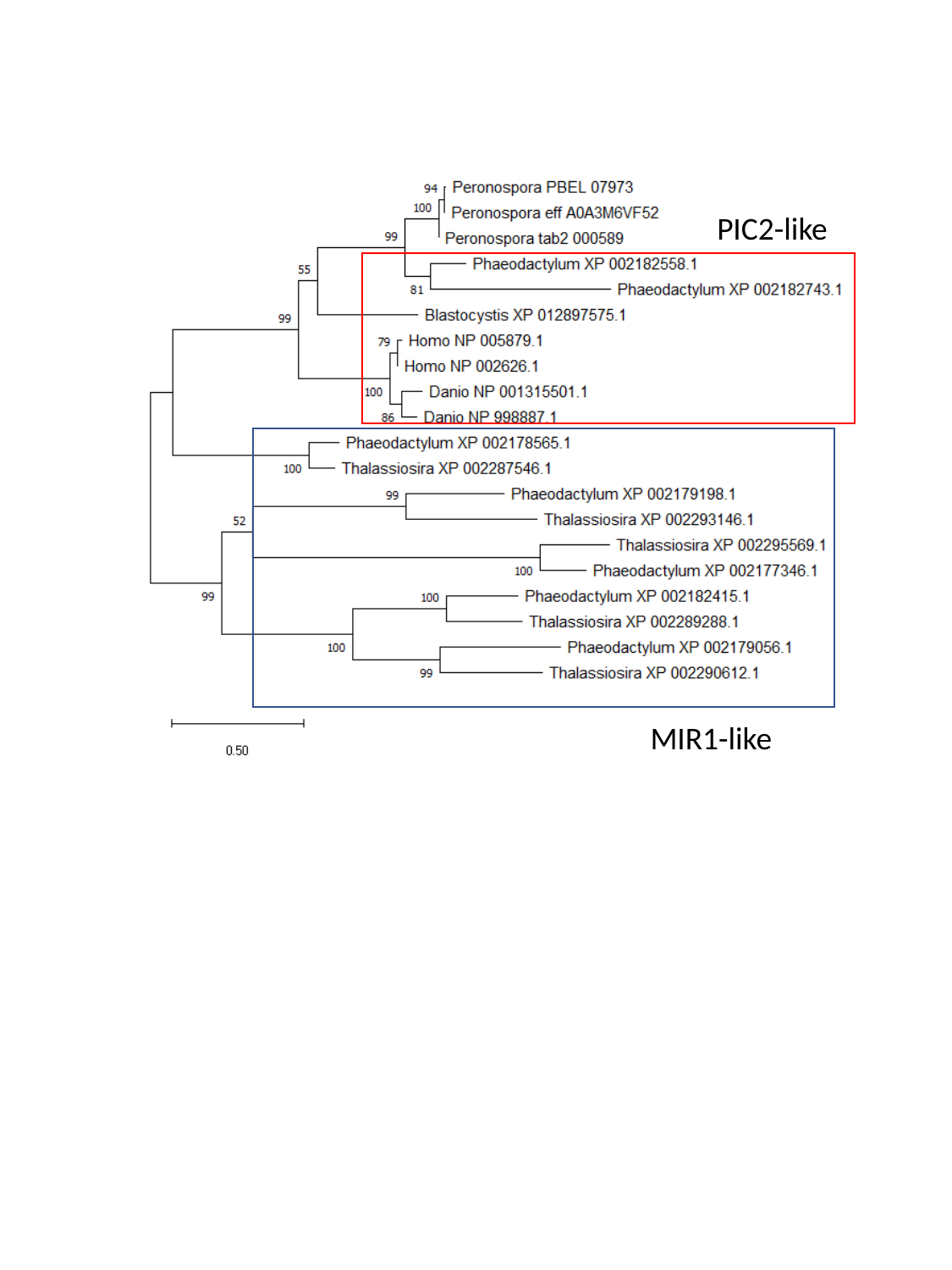

PIC2-like
MIR1-like

Supplement: S2 Fig — The maximum likelihood tree was constructed using the LG model. The percentage of replicate trees in which the associated proteins clustered together in the bootstrap test (1000 replicates) are posted next to the branches. The proteins in the red box are putative PIC2-like; the proteins in the blue box are putative MIR1-like transporters. The genus of the organism from which the protein originated and the Genbank number is listed on each branch; a ‘_’ follows each XP or NP. The bar indicates the number of protein substitutions per site. (PPTX) [file pone.0285685.s002.pptx]
