# Supplementary material for: A comparison of transporter gene expression in three species of Peronospora plant pathogens during host infection
Source: PLoS One. 2023 Jun 1;18(6):e0285685. doi: 10.1371/journal.pone.0285685 (PMC10234565; doi:10.1371/journal.pone.0285685)
Supplement: S2 File — (DOCX) [file pone.0285685.s009.docx]

CLUSTAL Omega (1.2.4) multiple sequence alignment

Ptab2_000589 ------------------------------------------------------------ 0

A0A3M6VF52 MMVLAKLRAGNALVEAAVRNSTAAFSMAGRVTFDEVTAGADVKQLEFMKEQIVQVDEQDY 60

PBEL_07973 ------------------------------------------------------------ 0

Ptab2_000589 ------------------------------------------------------------ 0

A0A3M6VF52 VVGPISKKDAHIHDGVLHRAFSVFVFNFENELLIQKRASEKITFPGFWANTCCSHPLFVE 120

PBEL_07973 ------------------------------------------------------------ 0

Ptab2_000589 ------------------------------------------------------------ 0

A0A3M6VF52 SELEDGVGVKRAAIRKLEHELGIPTSTFAINDLVYVSSVMYKAASGANWTEYEMDHILFA 180

PBEL_07973 ------------------------------------------------------------ 0

Ptab2_000589 ------------------------------------------------------------ 0

A0A3M6VF52 RGEVSLDKLNKNEVEQAEYVAHENLPTLLSDSTRKLSPWFHLIGSKLLPHWWSNLDTIFS 240

PBEL_07973 ------------------------------------------------------------ 0

Ptab2_000589 -MSAFFPVISTLNPAAPSNIVPKTPSTSILATSATKWDARLSHDPIHDTAYYGKCMIGGV 59

A0A3M6VF52 NMSTFFPVISTLNPAAPSIAAPKATSASVLATSAAKWDARLSHDPIHDTAYYGKCMIGGI 300

PBEL_07973 -MSALFPVISSLNPAAPPIAASKASSVSVLATSATKWDARLSHDPIHDNAYYGKCMIGGI 59

**::*****:****** . *: *.*:*****:*************.**********:

Ptab2_000589 LSCGLTHTGITPLDVVKCNMQVNPAKYNGLLPGLKTIASEEGAGALFKGWAPTAIGYSAQ 119

A0A3M6VF52 LSCGLTHTGITPLDVVKCNMQVNPAKYNGLLPGLKTIASEEGAGALFKGWAPTAIGYSAQ 360

PBEL_07973 LSCGLTHTGITPLDVVKCNMQVNPAKYNGLLPGLKTIASEEGAGALFKGWAPTAIGYSAQ 119

************************************************************

Ptab2_000589 GMCKFGFYEFFKDTYSTMVGEENAFKYRGAIYLAGSASAEFFADMALCPMEMVKVKVQTS 179

A0A3M6VF52 GMCKFGFYEFFKDTYSTMAGEENAYKYRGAIYLAGSASAEFFADMALCPMEMVKVKVQTS 420

PBEL_07973 GMCKFGFYEFFKDTYSTMAGEENAYKYRGAIYLAGSASAEFFADMALCPMEMVKVKVQTS 179

******************.*****:***********************************

Ptab2_000589 PAGTFPIEFGAAVAAMKANSAETRFPFGSLVPLWSRQIPYTMAKFFFFEKVVEAFYTYVF 239

A0A3M6VF52 PAGTFPVEFGAAVAAMKANSAETRFPFGSLVPLWSRQIPYTMAKFFFFEKVVEAFYTYVF 480

PBEL_07973 PAGTFPIEFGAAVAAMKANSAETRFPFGSLVPLWSRQIPYTMAKFFFFEKVVETFYTYVF 239

******:**********************************************:******

Ptab2_000589 TQPKSSYPKSTQLGVTFASGYLAGVICAIVSHPADSIVSLMGKAENKGKGFGQIASETGL 299

A0A3M6VF52 TEPKSSYPKSTQLGVTFASGYLAGVICAIVSHPADSVVSLMGKAENKGKGFGQIASETGL 540

PBEL_07973 TEPKSSYPKSTQLGVTFASGYLAGVICAIVSHPADSVVSLMGKAENKGKGFGQIASETGL 299

*:**********************************:***********************

Ptab2_000589 VNLATKGLGTRILMIGTLTGAQWWIYDTFKTVMGMGTSGGAASKKN 345

A0A3M6VF52 VNLATKGLGTRILMIGTLTGAQWWIYDTFKTVMGMGTSGGAAPKKN 586

PBEL_07973 VNLATKGLGTRIIMIGTLTGAQWWIYDTFKTVMGMGTSGGAAPKKN 345

************:***************************** ***
