# Supplementary material for: A comparison of transporter gene expression in three species of Peronospora plant pathogens during host infection
Source: PLoS One. 2023 Jun 1;18(6):e0285685. doi: 10.1371/journal.pone.0285685 (PMC10234565; doi:10.1371/journal.pone.0285685)
Supplement: S3 File — (DOCX) [file pone.0285685.s010.docx]

**Validation of Protein 3D Structure:** After modelling of structure, the protein structure was validated through the SAVES server (RAMPAGE (Ramachandran Plot Analysis), ERRAT, PROVE, and Verify3D).

|  | **Protein Codes** | **RAMPAGE** | **ERRAT** | **VERIFY 3D** | **PROVE** | **Predicted Ligand binding residues** | **Active site residues predicted by COFACTOR** |
| --- | --- | --- | --- | --- | --- | --- | --- |
| **Pair-1** | **>XP_011511389.1** | Favored regions-**85.8%**,  Additional allowed regions- **12.2%**,  Generously allowed regions- **1.1%**,  Disallowed regions- **0.9%** | Overall quality factor-**91.08** | **68.17%** of the residues have averaged 3D-1D score >= 0.2 | Mean Z-score- **0.206**  Z-score RMS- **1.550** | 43,47,178,  181,185,299,300,305,334,396,397,405,  432 | **159** active site residues, with C-score 0.154, TM-score is 0.348, and RMSD is 7.54 |
|  | **>A0A3R7W609** | Favored regions-**80.1%**  Additional allowed regions-**15.5%**  Generously allowed regions-**2.9%**  Disallowed regions-**1.5%** | Overall quality factor- **88.2453** | **73.95%** of the residues have averaged 3D-1D score >= 0.2 | Mean Z-score-**0.336**  Z-score RMS-**1.528** | 158,162,286,289,290,293,409,410,415,444,501,502,  510,537 | **162** active site residues, with C-score 0.127, TM-score is 0.337, and RMSD is 7.74 |
| **Pair-2** | **>NP_002626.1** | Favored regions-**77.1%**  Additional allowed regions-**19.0%**  Generously allowed regions-**1.6%**  Disallowed regions-**2.3%** | Overall quality factor-**87.535** | **70.91%** of the residues have averaged 3D-1D score >= 0.2 | Mean Z-score-  Z-score RMS- | 128,136,140,172,176,225,226,229,230,280,283,284,287 | **106** active site residues, with C-score 0.175, TM-score is 0.4, and RMSD is 5.83 |
|  | **>PBEL_07973_1** | Favored regions-**79.6%** Additional allowed regions-**14.9%**  Generously allowed regions-**3.1%**  Disallowed regions-**2.4%** | Overall quality factor-**92.8783** | **80.22%** of the residues have averaged 3D-1D score >= 0.2 | Mean Z-score-**0.273**  Z-score RMS-**1.523** | 115,123,127,159,163,215,216,219,220,270,273,274,277 | **25** active site residues, with C-score 0.165, TM-score is 0.372, and RMSD is 5.98 |
| **Pair-3** | **>A0A3M6VMF9** | Favored regions-**95.1%**  Additional allowed regions-**4.5%**  Generously allowed regions-**0.4%**  Disallowed regions-**0.0%** | Overall quality factor-**90.5797** | **72.82%** of the residues have averaged 3D-1D score >= 0.2 | Mean Z-score-**0.309**  Z-score RMS-**1.256** | 94,140,195,196,199,200,241,244,245,248 | NA |
|  | **>pdb\|1OKC\|A** | Favored regions-**95.2%**  Additional allowed regions-**4.0%**  Generously allowed regions-**0.8%**  Disallowed regions-**0.0%** | Overall quality factor-**91.5493** | **75.23%** of the residues have averaged 3D-1D score >= 0.2 | Mean Z-score-**0.273**  Z-score RMS-**1.255** | 79,87,91,123,127,182,183,186,187,227,230,231,234 | NA |

**Results:**

**Validation of Protein 3D Structure of >XP_011511389.1 and >A0A3R7W609 of PAIR 1:**

**1. ERRAT of >XP_011511389.1 and >A0A3R7W609 of PAIR 1:**


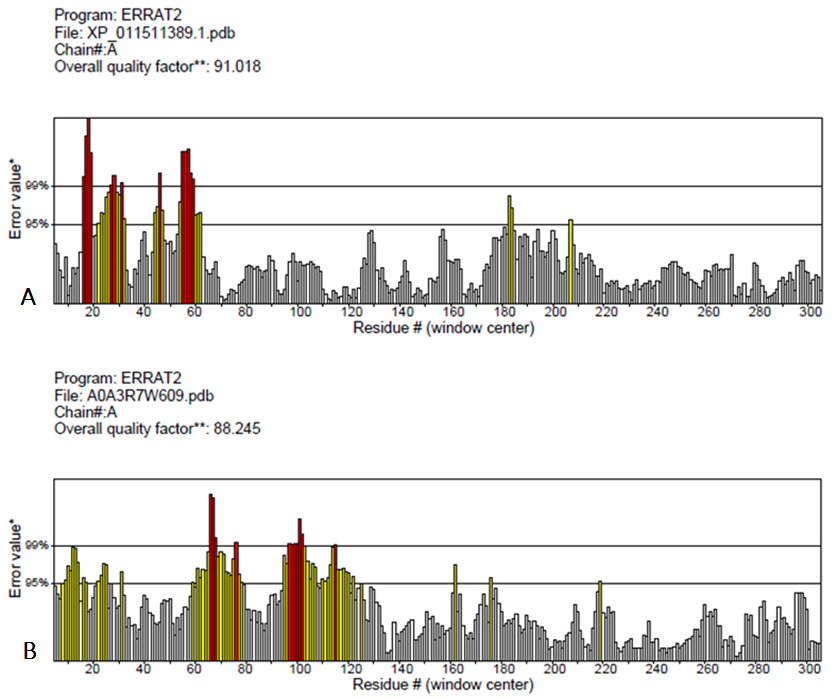


**Figure:** **Structure validation with ERRAT tool**. The result of ERRAT tool shows that overall quality factor of the **>XP_011511389.1** **(A)** and in **>A0A3R7W609 (B)** modelled protein based on various sorts of atoms were found to be 91.018 and 88.245 respectively which are satisfactory.

**PROVE (Protein Volume Evaluation) of >XP_011511389.1 and >A0A3R7W609 of PAIR 1:**


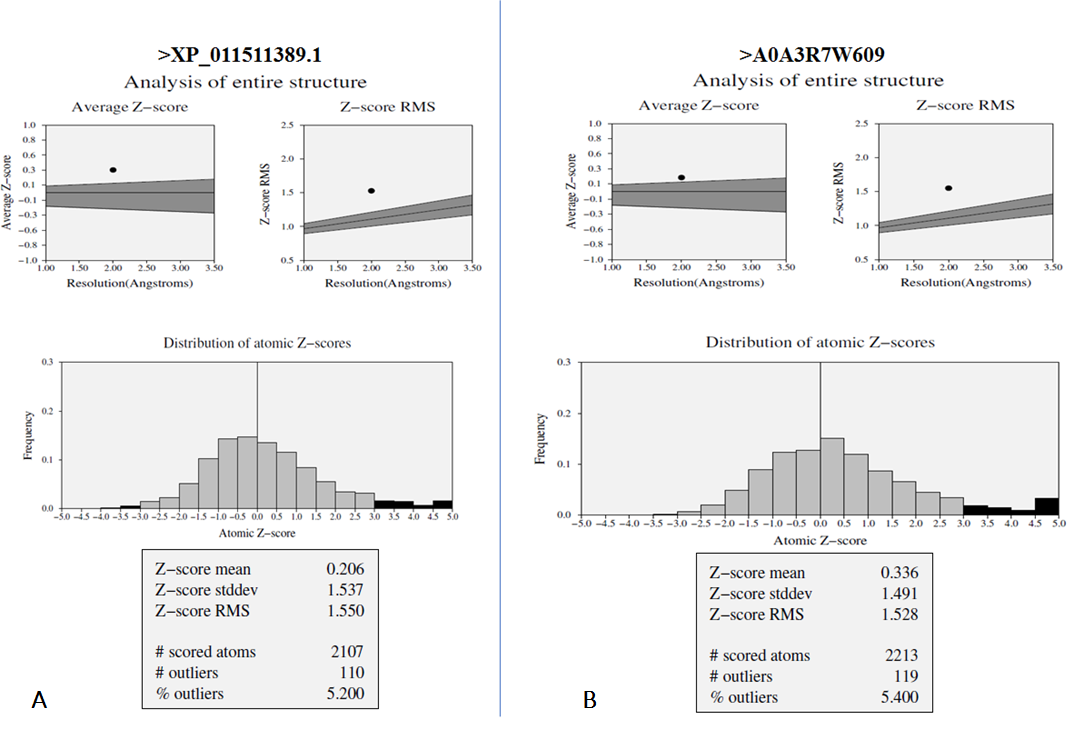


**Figure:** **PROVE analysis of protein**. Z-score defined the energy separation between native fold and average of an ensemble of misfold unit. **(A) >RMX65273_1 P T** has average Z-score was 0.206 and the Z-score RMS was 1.550 and (B) **>PITG_13003** has average Z-score was 0.336 and the Z-score RMS was 1.528

**Ramachandran Plot Analysis of >XP_011511389.1 and >A0A3R7W609 of PAIR 1:**


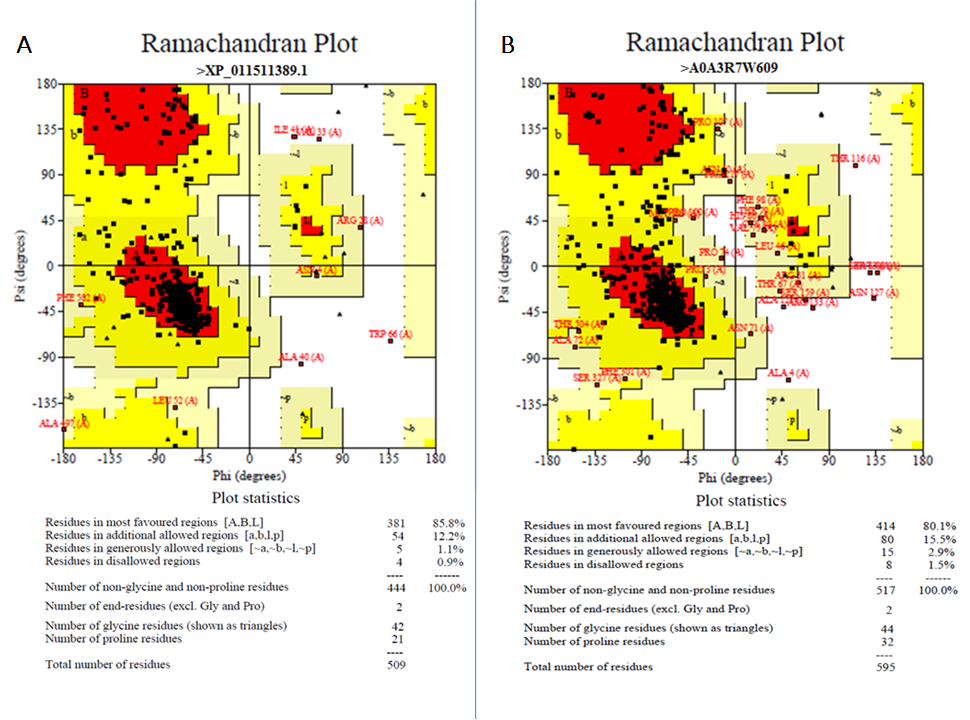


Figure: **Ramachandran plot analysis of modelled protein**. PROCHECK analysis shows that in favored regions, additional allowed region, generously allowed region, and disallowed region was 85.8%, 12.2%, 1.1% and 0.9% for **>XP_011511389.1** and 80.1%, 15.5%, 2.9%, and 1.5% for **>A0A3R7W609**, respectively.

**Structure Prediction of >XP_011511389.1 and >A0A3R7W609 of PAIR 1:**


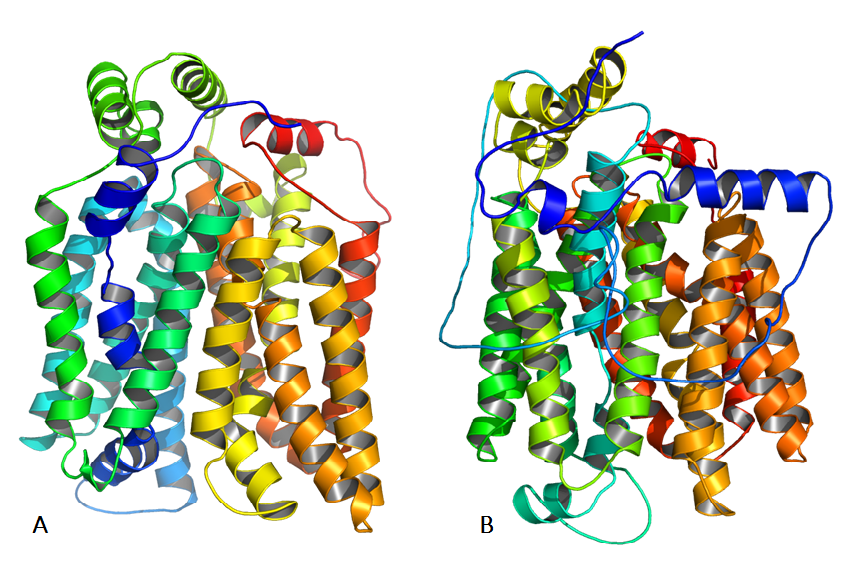


**Figure:** **Modelling of protein via I-TASSER: (A) >XP_011511389.1** protein modelled via I-TASSER showing C-score -0.14, estimated RMSD 7.7±4.3 Å, and estimated TM-score 0.70±0.12 and **(B)** **>A0A3R7W609** showing C-score -0.74, estimated RMSD 9.4±4.6 Å, and estimated TM-score 0.62±0.14.

**Structure alignment of >XP_011511389.1 and >A0A3R7W609 of PAIR 1:**


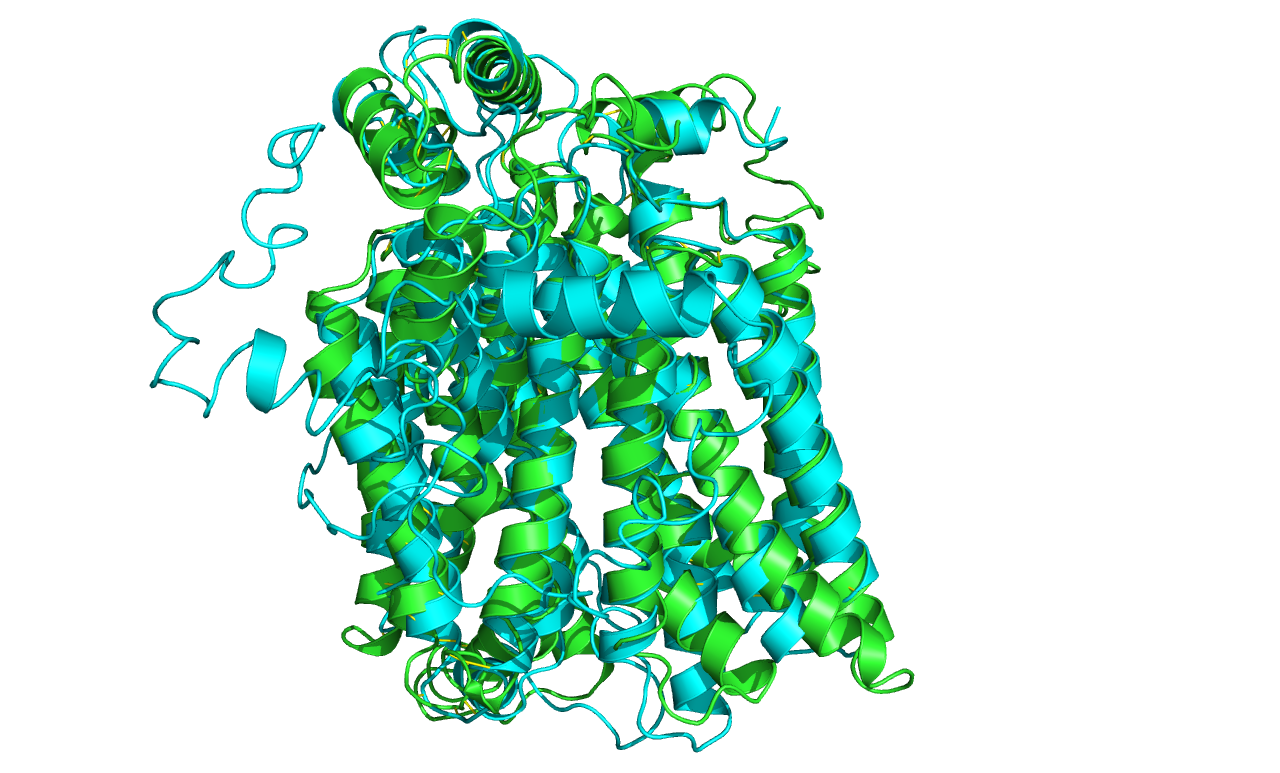


**Figure:** Aligned structure of modelled **>XP_011511389.1** (green) and **>A0A3R7W609** (cyan) of PAIR 1

**Validation of Protein 3D Structure of >NP_002626.1 and >PBEL_07973_1 of PAIR 2:**

**1. ERRAT of >NP_002626.1 and >PBEL_07973_1 of PAIR 2:**


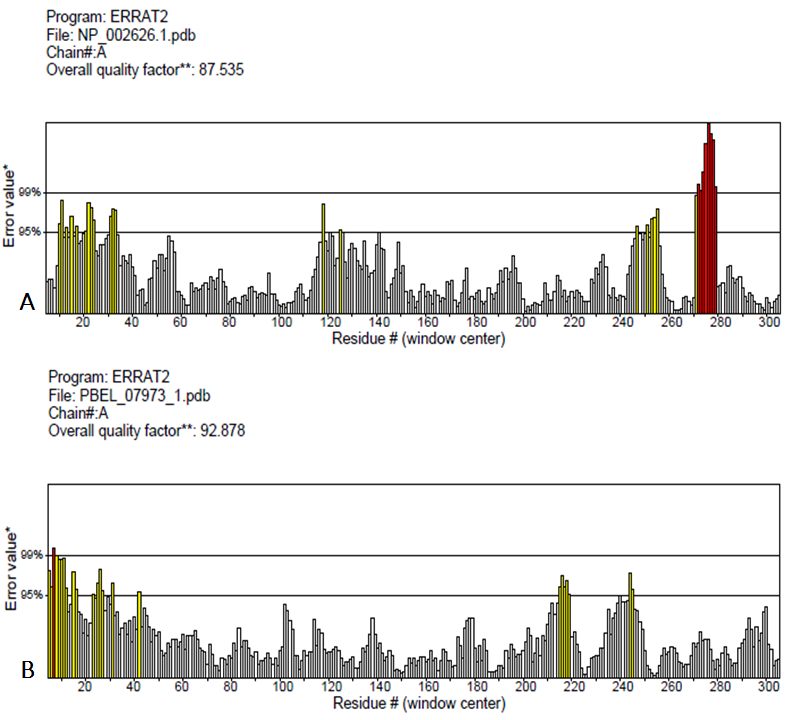


**Figure:** **Structure validation with ERRAT tool**. The result of ERRAT tool shows that overall quality factor of the **>NP_002626.1 (A)** and in **>PBEL_07973_1 (B)** modelled protein based on various sorts of atoms were found to be 87.535 and 92.878 respectively which are satisfactory.

**PROVE (Protein Volume Evaluation) of >NP_002626.1 and >PBEL_07973_1 of PAIR 2:**


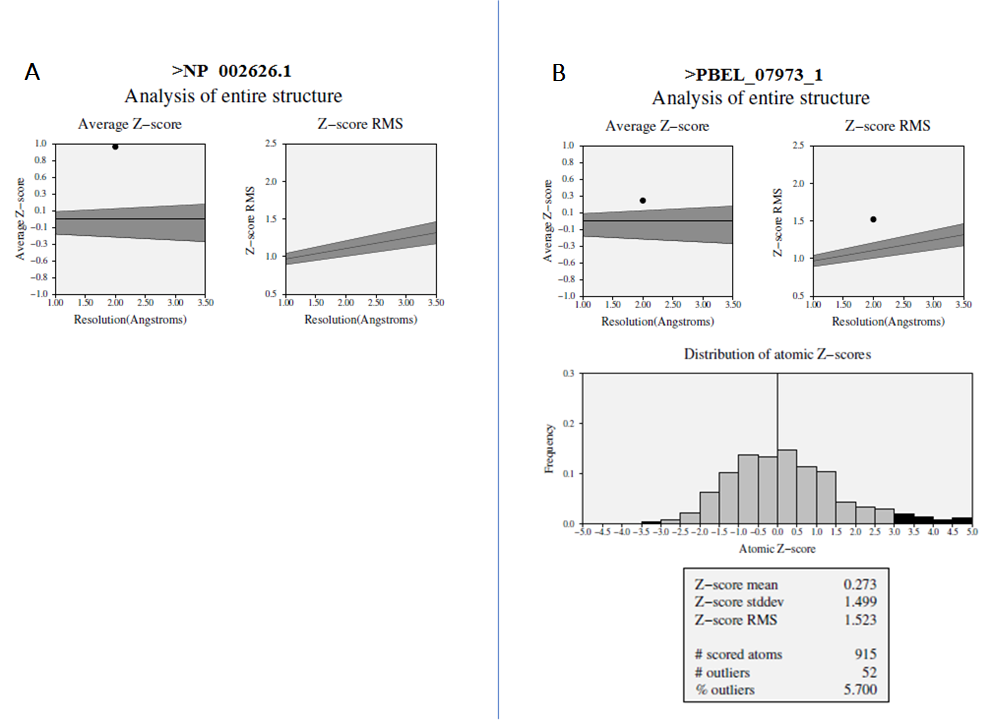


**Figure:** **PROVE analysis of protein**. Z-score defined the energy separation between native fold and average of an ensemble of misfold unit. **(A) >NP_002626.1** not determined and (B) **>PBEL_07973_1** has average Z-score was 0.273 and the Z-score RMS was 1.523

**Ramachandran Plot Analysis of >NP_002626.1 and >PBEL_07973_1 of PAIR 2:**


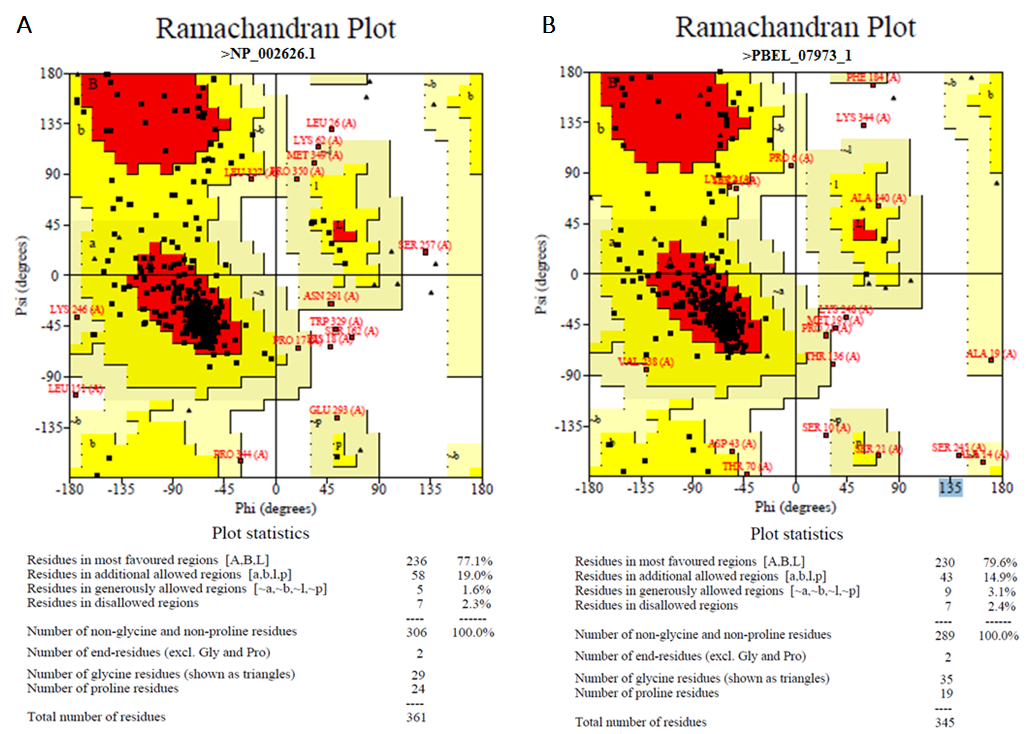


Figure: **Ramachandran plot analysis of modelled protein**. PROCHECK analysis shows that in favored regions, additional allowed region, generously allowed region, and disallowed region was 77.1%, 19.0%, 1.6% and 2.3% for **>NP_002626.1** and 79.6%, 14.9%, 3.1%, and 2.4% for **>PBEL_07973_1**, respectively.

**Structure Prediction of >NP_002626.1 and >PBEL_07973_1 of PAIR 2:**


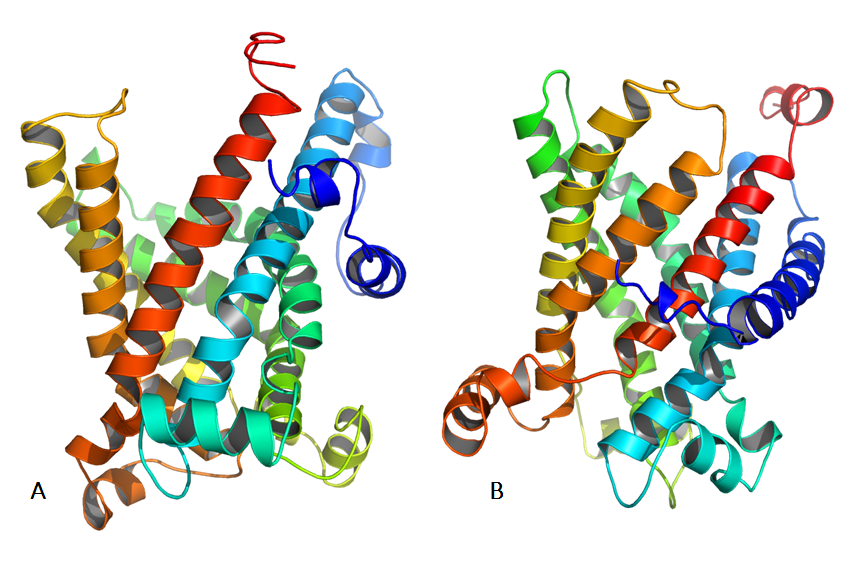


**Figure:** **Modelling of protein via I-TASSER: (A) >NP_002626.1** protein modelled via I-TASSER showing C-score -2.05, estimated RMSD 11.4±4.5 Å, and estimated TM-score 0.47±0.15 and **(B)** **>PBEL_07973_1** showing C-score -2.17, estimated RMSD 11.6±4.5 Å, and estimated TM-score 0.46±0.15.

**Structure alignment of >NP_002626.1 and >PBEL_07973_1 of PAIR 2:**


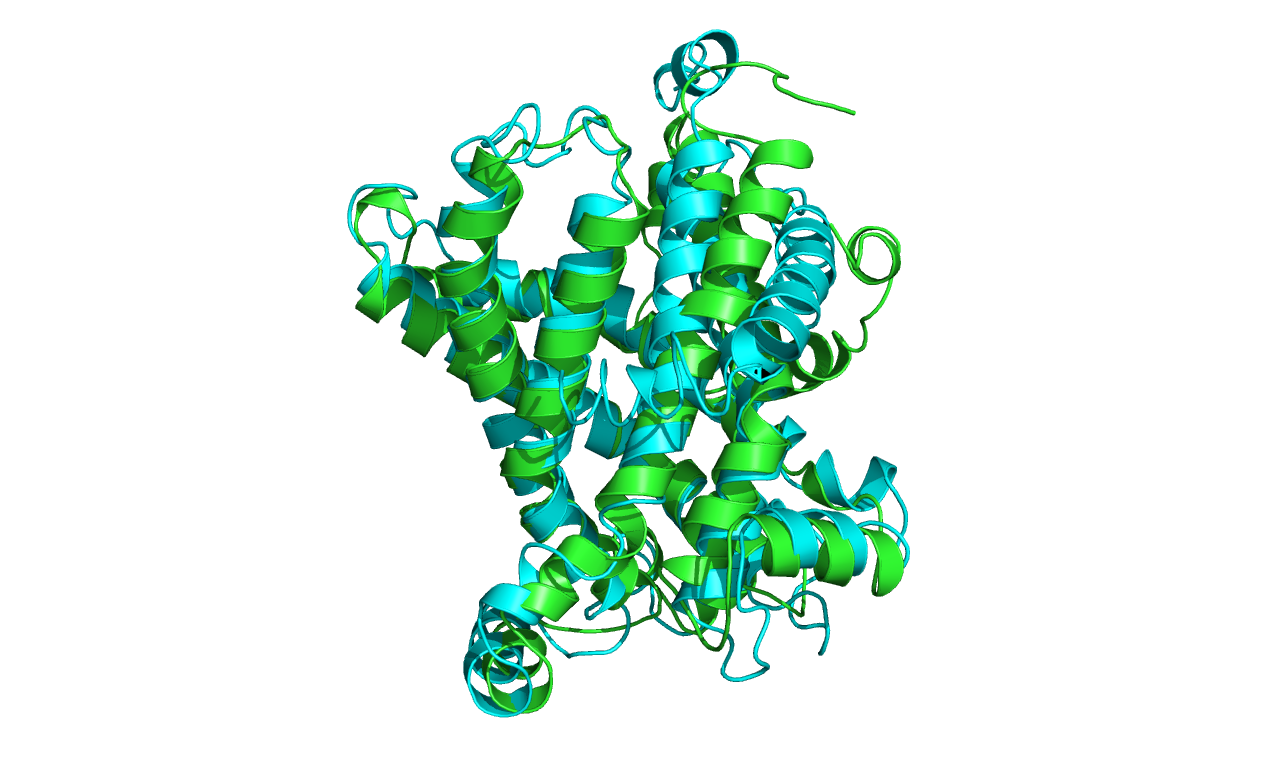


**Figure:** Aligned structure of modelled **>NP_002626.1** (green) and **>PBEL_07973_1** (cyan) of PAIR 2

**Validation of Protein 3D Structure of >A0A3M6VMF9 and >pdb|1OKC|A of PAIR 3:**

**1. ERRAT of >A0A3M6VMF9 and >pdb|1OKC|A of PAIR 3:**


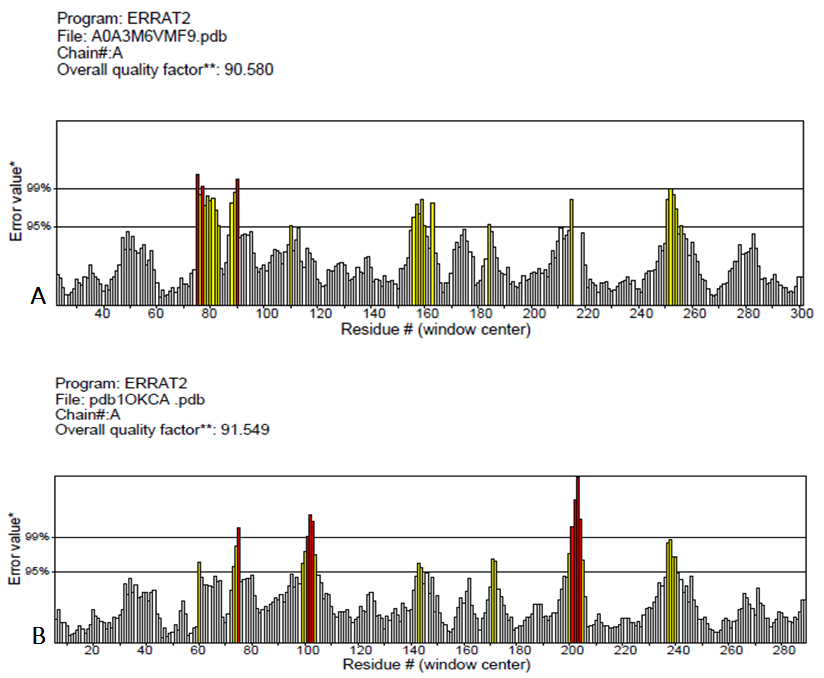


**Figure:** **Structure validation with ERRAT tool**. The result of ERRAT tool shows that overall quality factor of the **>A0A3M6VMF9 (A)** and in **>pdb|1OKC|A (B)** modelled protein based on various sorts of atoms were found to be 90.580 and 91.549 respectively which are satisfactory.

**PROVE (Protein Volume Evaluation) of >A0A3M6VMF9 and >pdb|1OKC|A of PAIR 3:**


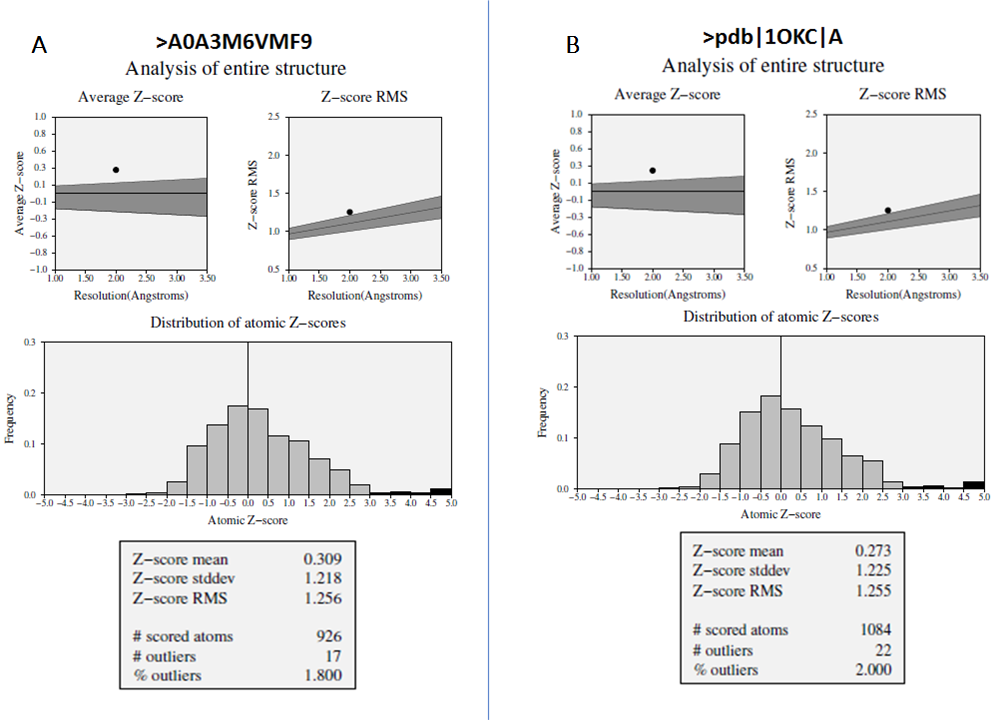


**Figure:** **PROVE analysis of protein**. Z-score defined the energy separation between native fold and average of an ensemble of misfold unit. **(A) >A0A3M6VMF9** has average Z-score was 0.309 and the Z-score RMS was 1.256 and (B) **>pdb|1OKC|A** has average Z-score was 0.273 and the Z-score RMS was 1.255

**Ramachandran Plot Analysis of >A0A3M6VMF9 and >pdb|1OKC|A of PAIR 3:**


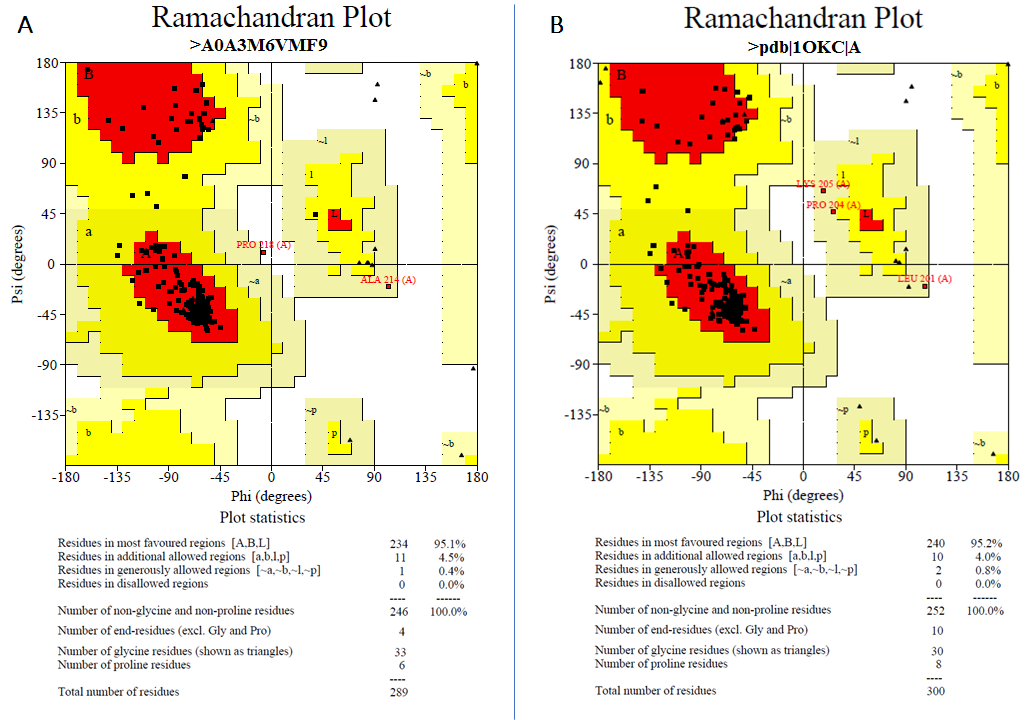


Figure: **Ramachandran plot analysis of modelled protein**. PROCHECK analysis shows that in favored regions, additional allowed region, generously allowed region, and disallowed region was 95.1%, 4.5%, 0.4% and 0.0% for **>A0A3M6VMF9** and 95.2%, 4.0%, 0.8%, and 0.0% for **>pdb|1OKC|A**, respectively.

**Structure Prediction of >A0A3M6VMF9 and >pdb|1OKC|A of PAIR 3:**


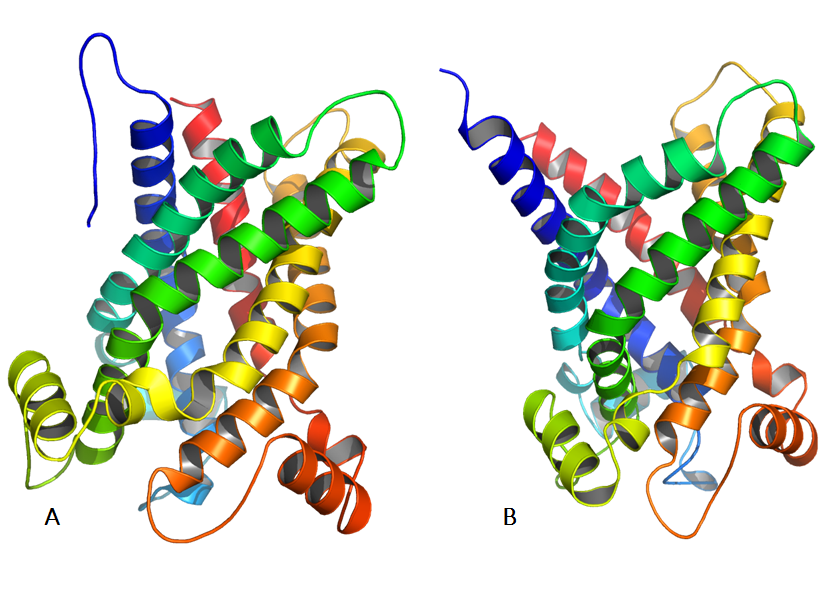


**Figure:** **Modelling of protein via I-TASSER: (A) >A0A3M6VMF9** protein modelled via I-TASSER showing C-score -0.13, estimated RMSD 6.5±3.9 Å, and estimated TM-score 0.70±0.12 and **(B)** **>pdb|1OKC|A** showing C-score 1.69, estimated RMSD 2.8±2.0 Å, and estimated TM-score 0.95±0.05.

**Structure alignment of >A0A3M6VMF9 and >pdb|1OKC|A of PAIR 3:**


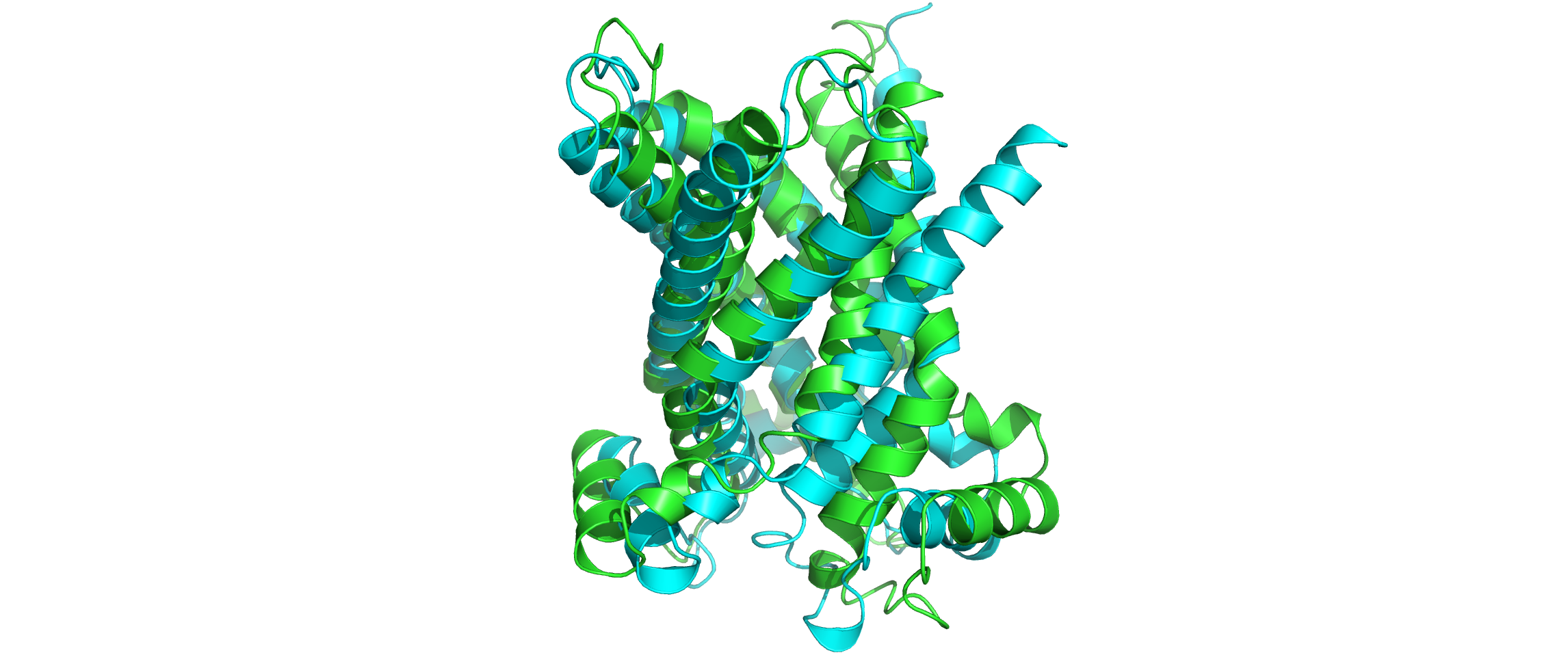


**Figure:** Aligned structure of modelled **>A0A3M6VMF9** (green) and **>pdb|1OKC|A** (cyan) of PAIR 3.

TM-align is a protein structural comparison algorithm that is sequence agnostic. Using systematic dynamic programming cycles, TM-align first creates optimum residue-to-residue match for two protein structures with undetermined structural equivalency. In addition to the TM-score value, which scales the structural similarity, an ideal superposition of the two structures based on the observed alignment will be provided. Then TM-score ranges from 0 to 1, with 1 denoting a good matching score between the two structures. Scores below 0.2 belong to randomly selected unrelated proteins, according to stringent statistics of structures in the PDB, whereas scores over 0.5 are often the same fold in SCOP (Structural Classification of Proteins)/ CATH (Class, Architecture, Topology, and Homology Protein Structure Database).

**FOR PROTEINS OF PAIR-1**

**Length of >XP_011511389.1:** 509 residues

**Length of >A0A3R7W609:** 595 residues

**Aligned length =** 455, **RMSD =** 2.72 (of two aligned structures)

**Sequence Identity =** No. of identical residues/ Total aligned residues * 100 = 30.5%

TM-score = 0.82764 (if normalized by length of **>XP_011511389.1)**

TM-score = 0.82764 (if normalized by length of **>A0A3R7W609)**

**FOR PROTEINS OF PAIR-2**

**Length of >NP_002626.1:** 361 residues

**Length of >PBEL_07973_1:** 345 residues

**Aligned length =** 303, **RMSD =** 3.16 (of two aligned structures)

**Sequence Identity =** No. of identical residues/ Total aligned residues * 100 = 47.9%

TM-score = 0.73614 (if normalized by length of **>NP_002626.1)**

TM-score = 0.76736 (if normalized by length of **>PBEL_07973_1)**

**FOR PROTEINS OF PAIR-3**

**Length of >A0A3M6VMF9:** 309 residues

**Length of >pdb|1OKC|A Chain A:** 297 residues

**Aligned length =** 289, **RMSD =** 4.41 (of two aligned structures)

**Sequence Identity =** No. of identical residues/ Total aligned residues * 100 = 57.8%

**TM-score =** 0.68872 (if normalized by length of **>A0A3M6VMF9)**

**TM-score =** 0.71108 (if normalized by length of **>pdb|1OKC|A Chain A)**

**Note:** As the TM-score moves closer to 1 on the scale from 0 to 1, indicates the good alignment between the two aligned structures. When compare the two aligned structures, RMSD is the most often reported metric, however it is susceptible to the local structural deviance. Even when the remainder of the structure is properly aligned, the RMSD value will be high if a few residues in a loop are poorly aligned. So, TM-align program is more reliable to predict the goodness of two aligned protein structures.

**References:**

Y. Zhang, J. Skolnick, TM-align: A protein structure alignment algorithm based on TM-score, Nucleic Acids Research, 33: 2302-2309 (2005)
