# Supplementary material for: A comparison of transporter gene expression in three species of Peronospora plant pathogens during host infection
Source: PLoS One. 2023 Jun 1;18(6):e0285685. doi: 10.1371/journal.pone.0285685 (PMC10234565; doi:10.1371/journal.pone.0285685)
Supplement: Attachment — Submitted filename: Response to Reviewers.docx [file pone.0285685.s011.docx]

PONE-D-22-25502

A comparison of transporter gene expression in three species of Peronospora plant pathogens during host infection

PLOS ONE

Dear Dr. Johnson,

Thank you for submitting your manuscript to PLOS ONE. You may confirm that both reviewers acknowledged the quality of your study, and I agree on them, but raised very constructively a number of important issues that need to be addressed before publication. Therefore, we invite you to submit a revised version of the manuscript that addresses the points raised during the review process.

Please submit your revised manuscript by Jan 12 2023 11:59PM. If you will need more time than this to complete your revisions, please reply to this message or contact the journal office at [plosone@plos.org](mailto:plosone@plos.org). When you're ready to submit your revision, log on to <https://www.editorialmanager.com/pone/> and select the 'Submissions Needing Revision' folder to locate your manuscript file.

Please include the following items when submitting your revised manuscript:

- A rebuttal letter that responds to each point raised by the academic editor and reviewer(s). You should upload this letter as a separate file labeled 'Response to Reviewers'.
- A marked-up copy of your manuscript that highlights changes made to the original version. You should upload this as a separate file labeled 'Revised Manuscript with Track Changes'.
- An unmarked version of your revised paper without tracked changes. You should upload this as a separate file labeled 'Manuscript'.

If you would like to make changes to your financial disclosure, please include your updated statement in your cover letter. Guidelines for resubmitting your figure files are available below the reviewer comments at the end of this letter.

If applicable, we recommend that you deposit your laboratory protocols in protocols.io to enhance the reproducibility of your results. Protocols.io assigns your protocol its own identifier (DOI) so that it can be cited independently in the future. For instructions see: [https://journals.plos.org/plosone/s/submission-guidelines#loc-laboratory-protocols](https://gcc02.safelinks.protection.outlook.com/?url=https%3A%2F%2Fjournals.plos.org%2Fplosone%2Fs%2Fsubmission-guidelines%23loc-laboratory-protocols&data=05%7C01%7C%7C148dee5cd8ca4bf0dc6908dad154138d%7Ced5b36e701ee4ebc867ee03cfa0d4697%7C0%7C0%7C638052456770993665%7CUnknown%7CTWFpbGZsb3d8eyJWIjoiMC4wLjAwMDAiLCJQIjoiV2luMzIiLCJBTiI6Ik1haWwiLCJXVCI6Mn0%3D%7C3000%7C%7C%7C&sdata=fEAZw1NSyudoIY3uHLT0K5sV2uvmvO4vihdOKGsUr50%3D&reserved=0). Additionally, PLOS ONE offers an option for publishing peer-reviewed Lab Protocol articles, which describe protocols hosted on protocols.io. Read more information on sharing protocols at [https://plos.org/protocols?utm_medium=editorial-email&utm_source=authorletters&utm_campaign=protocols](https://gcc02.safelinks.protection.outlook.com/?url=https%3A%2F%2Fplos.org%2Fprotocols%3Futm_medium%3Deditorial-email%26utm_source%3Dauthorletters%26utm_campaign%3Dprotocols&data=05%7C01%7C%7C148dee5cd8ca4bf0dc6908dad154138d%7Ced5b36e701ee4ebc867ee03cfa0d4697%7C0%7C0%7C638052456770993665%7CUnknown%7CTWFpbGZsb3d8eyJWIjoiMC4wLjAwMDAiLCJQIjoiV2luMzIiLCJBTiI6Ik1haWwiLCJXVCI6Mn0%3D%7C3000%7C%7C%7C&sdata=a1t4%2BQRFlW2MLz%2BENUqyQYwjj3U1%2FNEYjVq2xfyFjA0%3D&reserved=0).

We look forward to receiving your revised manuscript.

Kind regards,

Hernâni Gerós, PhD
Academic Editor
PLOS ONE

Journal Requirements:

When submitting your revision, we need you to address these additional requirements.

1. Please ensure that your manuscript meets PLOS ONE's style requirements, including those for file naming. The PLOS ONE style templates can be found at

<https://journals.plos.org/plosone/s/file?id=wjVg/PLOSOne_formatting_sample_main_body.pdf> and

[https://journals.plos.org/plosone/s/file?id=ba62/PLOSOne_formatting_sample_title_authors_affiliations.pdf](https://gcc02.safelinks.protection.outlook.com/?url=https%3A%2F%2Fjournals.plos.org%2Fplosone%2Fs%2Ffile%3Fid%3Dba62%2FPLOSOne_formatting_sample_title_authors_affiliations.pdf&data=05%7C01%7C%7C148dee5cd8ca4bf0dc6908dad154138d%7Ced5b36e701ee4ebc867ee03cfa0d4697%7C0%7C0%7C638052456770993665%7CUnknown%7CTWFpbGZsb3d8eyJWIjoiMC4wLjAwMDAiLCJQIjoiV2luMzIiLCJBTiI6Ik1haWwiLCJXVCI6Mn0%3D%7C3000%7C%7C%7C&sdata=ulgs0UYAIZ%2B7F4rSexuXT4D%2Fm%2FyXE4%2FMlfJ2%2FaRcbG0%3D&reserved=0)

2. Please expand the acronym “USDA ARS” (as indicated in your financial disclosure) so that it states the name of your funders in full.

This information should be included in your cover letter; we will change the online submission form on your behalf.

3.  Please include captions for your Supporting Information files at the end of your manuscript, and update any in-text citations to match accordingly. Please see our Supporting Information guidelines for more information: [http://journals.plos.org/plosone/s/supporting-information](https://gcc02.safelinks.protection.outlook.com/?url=http%3A%2F%2Fjournals.plos.org%2Fplosone%2Fs%2Fsupporting-information&data=05%7C01%7C%7C148dee5cd8ca4bf0dc6908dad154138d%7Ced5b36e701ee4ebc867ee03cfa0d4697%7C0%7C0%7C638052456770993665%7CUnknown%7CTWFpbGZsb3d8eyJWIjoiMC4wLjAwMDAiLCJQIjoiV2luMzIiLCJBTiI6Ik1haWwiLCJXVCI6Mn0%3D%7C3000%7C%7C%7C&sdata=Z7O8iDA6ZueXM3bcVtYMWfy9IdZ%2FpWOydHQFBlqgmzA%3D&reserved=0).

4. Please upload a new copy of Figures 1, 2, 3 and 4 as the detail is not clear. Please follow the link for more information: <https://blogs.plos.org/plos/2019/06/looking-good-tips-for-creating-your-plos-figures-graphics/>" [https://blogs.plos.org/plos/2019/06/looking-good-tips-for-creating-your-plos-figures-graphics/](https://gcc02.safelinks.protection.outlook.com/?url=https%3A%2F%2Fblogs.plos.org%2Fplos%2F2019%2F06%2Flooking-good-tips-for-creating-your-plos-figures-graphics%2F&data=05%7C01%7C%7C148dee5cd8ca4bf0dc6908dad154138d%7Ced5b36e701ee4ebc867ee03cfa0d4697%7C0%7C0%7C638052456770993665%7CUnknown%7CTWFpbGZsb3d8eyJWIjoiMC4wLjAwMDAiLCJQIjoiV2luMzIiLCJBTiI6Ik1haWwiLCJXVCI6Mn0%3D%7C3000%7C%7C%7C&sdata=t1fxTIICIgVdnd%2BmlqblQ1G0S0MoCOB46j6TKglTGdg%3D&reserved=0)

[Note: HTML markup is below. Please do not edit.] To

Reviewers' comments:

Reviewer's Responses to Questions

**Comments to the Author**

1. Is the manuscript technically sound, and do the data support the conclusions?

The manuscript must describe a technically sound piece of scientific research with data that supports the conclusions. Experiments must have been conducted rigorously, with appropriate controls, replication, and sample sizes. The conclusions must be drawn appropriately based on the data presented.

Reviewer #1: Partly

Reviewer #2: Yes

2. Has the statistical analysis been performed appropriately and rigorously?

Reviewer #1: No

Reviewer #2: Yes

3. Have the authors made all data underlying the findings in their manuscript fully available?

The [PLOS Data policy](https://gcc02.safelinks.protection.outlook.com/?url=http%3A%2F%2Fwww.plosone.org%2Fstatic%2Fpolicies.action%23sharing&data=05%7C01%7C%7C148dee5cd8ca4bf0dc6908dad154138d%7Ced5b36e701ee4ebc867ee03cfa0d4697%7C0%7C0%7C638052456770993665%7CUnknown%7CTWFpbGZsb3d8eyJWIjoiMC4wLjAwMDAiLCJQIjoiV2luMzIiLCJBTiI6Ik1haWwiLCJXVCI6Mn0%3D%7C3000%7C%7C%7C&sdata=IwVibISj3nsoHazVODASQvYzA5f0Q2d%2FT8qObC386KY%3D&reserved=0) requires authors to make all data underlying the findings described in their manuscript fully available without restriction, with rare exception (please refer to the Data Availability Statement in the manuscript PDF file). The data should be provided as part of the manuscript or its supporting information, or deposited to a public repository. For example, in addition to summary statistics, the data points behind means, medians and variance measures should be available. If there are restrictions on publicly sharing data—e.g. participant privacy or use of data from a third party—those must be specified.

Reviewer #1: Yes

Reviewer #2: Yes

4. Is the manuscript presented in an intelligible fashion and written in standard English?

PLOS ONE does not copyedit accepted manuscripts, so the language in submitted articles must be clear, correct, and unambiguous. Any typographical or grammatical errors should be corrected at revision, so please note any specific errors here.

Reviewer #1: No

Reviewer #2: Yes

5. Review Comments to the Author

Please use the space provided to explain your answers to the questions above. You may also include additional comments for the author, including concerns about dual publication, research ethics, or publication ethics. (Please upload your review as an attachment if it exceeds 20,000 characters)

Reviewer #1: Dear Authors,

Your Plos1 report on Peronospora transcriptomics was extended for my peer-review. In it, you generate RNAseq data for P. tabacina and re-analyze similar data from two related pathogens. Your study is a report on bioinformatic analyses with virtually no wet-lab studies beyond these required for the generation of RNAseq data. As such, this leaves me wanting for more rigorous research, to substantiate your conclusions. Writing of this report is generally acceptable, but I flag below numerous issues, with those indicated as [MAJOR] being of particular importance, in my opinion.

l.22 onto their hosts {specify} Changed to: onto leaves of their hosts. The hosts are specified in the fourth sentence of the abstract, so we do not think it is necessary to name the hosts again.

l.22-24 How measured? Changed to: measured using RNA sequencing.

l/43 vs. l.50 [MAJOR] Phytopathologically, one cannot control/manage a disease. Disease is the result (signs and symptoms) of compatible interaction between a pathogen and a (susceptible) plant host. Phrases as in l.50 or "resistance to downy mildew" l.50-51 are mental shortcuts, not to be used in technical, precise, scientific writing. Furthermore, Authors DO use occasionally the correct phrasing: l.56 "pathogen is controlled". Please unify/correct throughout.

Resistance to downy mildew, or similar phrase, changed throughout the manuscript.

52 resistant -> resistance changed as recommended
60 while -> whereas {correct throughout} corrected throughout the manuscript
71 while -> Although changed as recommended
87 'that encode... carbohydrate' please rephrase for clarity/meaning this sentence was edited
99 [MAJOR] Be specific. This paragraph and those that follow actually only describe P. tabacina processing. The subtitle on lines 99 and 114 were modified to be more specific
122 Describe briefly the RNA isolation/quality checks; do not make readers look in other sources. A brief description of extraction of RNA from leaves was added.
130-131 CITE if published a citation was added
140 see comment above a citation was added
152 [MAJOR] Describe what was used as house-keeping gene(s) incl. GenBank seq numbers and citations, if published. An additional supplementary file was added that contained all the sequences of the housekeeping genes; for this reason GenBank sequence numbers were not included in the supplementary file. Also add, what sample(s) served as baseline. No samples served as a baseline in this study; gene expression in each pathogen at each timepoint was analyzed independently.
154 Proteins -> Amino acid sequences (if that was the intended meaning). "Protein alignments" -> Sequence alignments the. Changed as recommended.
[MAJOR] Details of this analysis missing : What was the mutation model/matrix used, and how was it chosen? Was parsimony correction implemented? What was the outgroup / root and why? How many bootstraps used? These details are written in the figure legends. What was the software (add version and citation)? The software program MEGA version 10 was listed and cited in this section of the Materials and Methods.
161 {in the range of} Changed as recommended.
162 high{er} if Changed as recommended.
166-167 [MAJOR] Please provide more substantiation for this focus. We think that the last paragraph of the introduction discusses why we chose to analyze gene expression of transporters.
173-174 was not statistically significant -> lacked statistical support. Changed as recommended.
181 significant number (How was the significance of this result assessed? This number is comparable to MFS). We agree that the use of ‘significant’ is not appropriate; therefore this word was removed.
185 measured -> assessed/analyzed(?). We substituted ‘analyzed’ for ‘measured’.

188-189 significant expression {Again, the rationale/test data for the significance claim are missing; What was the significance threshold? How established?}. We substituted ‘enhanced’ for ‘significant’.
190 "Many more..." please append with the respective data point (Table/Figure). The data are in Supplementary File 3, which was noted in the sentence.
190-191 [MAJOR] This sentence rather indicates higher expression levels than more genes expressed. What other analyses / results can support the original claim? This sentence was edited for more clarity.
200 "The same transporter genes were measured in RNA samples" Imprecise. Please detail what was done and how. More detail was added to this sentence; the ‘how’ is described in the materials and methods section.
210 {expression levels of} the PBEL_08012... [drop the TPM]. Changed as suggested.
225 and 234 [MAJOR] the top 50 genes / 45 genes {By what feature? Same feature in both cases?}. The sentence describing the top 50 and top 45 most highly expressed genes was edited for clarity.
236 Consider adding more broad-scale context, as available in the following papers: 10.1093/gbe/evz048 ; 10.1016/j.fgb.2006.07.005 ; 10.1016/bs.adgen.2020.03.001 ; 10.1094/PHYTO-03-21-0092-R ; 10.1146/annurev-phyto-102313-050056 ; 10.1080/07352689.2018.1530848 ; 10.1111/nph.16092 ; 10.1094/phyto-05-15-0127-rvw ; 10.1101/2020.02.12.941195. A few more sentences were added at this point to provide additional context.
243 (30) Other great resources to add context are: 10.1111/mpp.12263 ; 10.1111/mpp.12260. A few sentences were added to describe the biotrophic phase of P. infestans.
291 Curiously (?) Consider rephrasing. ‘Curiously’ was removed.
297 CTL [Abbreviation not introduced]. Abbreviation was defined
312 [MAJOR] substance {A major offence to cell biology. Consider replacing with "structure" or similar}. ‘Structure’ was used instead of ‘substance’.
335 well -> highly. Changed as suggested.
340-345 [MAJOR] Methodological details of all that are missing in M&M. We used Clustal omega for the proteins sequence alignment; this analysis was described in the materials and methods. We changed the manuscript text at line 340 to make this more clear. We added more details about the mapping analysis to the materials and methods section.
353 [MAJOR] HMMTOP and TMHMM are missing from M&M. Details of this analysis, including that for SPLIT 4.0, were added to the materials and methods.
372 [MAJOR] TM-align details are missing from M&M. Details of this analysis were added to the materials and methods.
382-383 [MAJOR] Details missing from M&M. Details of this analysis were added to the materials and methods.
406 discover -> uncover. Changed as suggested.
414 Most -> Majority. Changed as suggested.
417 carbohydrate{s}. Changed as suggested.
434 [MAJOR] Consider discussing the context of P.tabacina fungicide resistance, e.g. 10.1094/PHYTO-03-21-0092-R ; 10.3390/agronomy5040555 and the older, descriptive studies We think that a discussion about fungicide resistance in P. tabacina is moving away from the primary topic of the manuscript.

442 [MAJOR] downy mildews -> pathogens. Changed as suggested.
453;454 firm -> company. Added (companies) to the sentence.
459 But (?). Removed ‘But’.
462 KhanC (?). Removed the superscript.

Reviewer #2: Dear editor and authors,

The authors developed in this study a comparison of gene expression of transporter proteins of three different Peronospora species during infection. The manuscript is simple, clear, well structured and written. For the study, they performed the infection experiments for one species and used two different public databases of published experiments. Therefore, this study is partially based on original scientific research but the analysis of the public databases is interesting. The standard of the experiments is of high degree and the methods are sufficiently detailed, as well as the statistical analysis. The conclusions are based in the results and are well presented.

In the next line I present a series of recommendations and questions to debate with the authors.
• Line 26: This type of discussion is not adequate for the abstract. This specific line is a summary of our data in two different sentences. These data are mentioned in more detail in the discussion section.
• Why the authors blasted the putative transporters of P. belbahrii and P. tabacina from the P. effusa ones and not from the paper that you identified the P. effusa ones? Kandel et al. did transcriptome analysis of spinach infected with P. effusa, however they did not identify any putative transporter genes in P. effusa. The transporter proteins of P. effusa were predicted from its genome sequence and downloaded from the InterPro website. These putative transporter proteins were checked for a statistically significant transporter motif and then the quality checked protein sequences were used to identify the putative transporters of P. belbahrii and P. tabacina.
• What is the rational of study these 3 pathogen species? Downy mildew disease causes major losses on economically important plants, such as those in this study; it is important to find out more detail about how these pathogens infect their hosts. In addition, we thought it would be of interest to analyze transporter gene expression in these three pathosystems to see if there are similarities or differences in the most highly expressed transporter genes during host infection.
• Can the authors explain the discrepancies regarding the days after inoculation of the samples? The studies of the basil, spinach and tobacco were independently completed. However, we felt that we could analyze this data together in this paper since the early timepoint (2 or 3 DPI) or the late timepoint (5, 6, or 7 DPI) only deviated one day or le ss than one day.
• Are not other databases using P. effusa pathogenesis that can be used because of first point of infection? We are not aware of additional P. effusa-spinach transcriptome data that is publicly available. In other words, we could only find one set of P. effusa-spinach transcriptome data.
• I cannot understand the value of the bars graphs presented in figure 1 without the names of the genes. We think it would be difficult to include the names of 50 genes on the horizontal axis. Readers would be able to find the gene name using the supplementary tables S3-S5.
• Line 197: “In addition, the TPM levels of two MFS, two MC and one Amino Acid/Auxin Permease (AAAP) gene were close to the same TPM levels of the housekeeping genes tubulin B and pyruvate 199 dehydrogenase alpha subunit in P. effusa at 7 DPI (Table 2).” Why is this relevant? We think this is relevant because we identified two transporter genes with expression levels close to the expression levels of important housekeeping genes. This suggests that these two transporter genes have an important function in the pathogen.
• The gene code presented in table 1 is the “same” for homologous genes? Is confusing the nomenclature in the text differs from the table. Maybe you can opt to introduce the specie gene name and its homologue. Admittedly it was difficult to identify a system for naming homologous genes. We settled on using the P. effusa RMX or RQM number as the homologous gene identifier in each Peronospora species. Readers can notice that the RMX/RQM naming system is used in Tables 2-4 for cross comparison. We think that we went through the Tables sequentially in the manuscript and introduce protein designations in the text (which were species specific) as needed. Moreover, Reviewer #1 did not mention a problem with our homologous gene identifiers.
• Line 215: “The TPM level of the MC gene PBEL_00715, as well as that of the actin gene, decreased from 3 216 to 6 DPI.” Why is this line relevant? Are these the only genes that decrease from 3 to 6 dpi? We thought it was interesting to find the expression (TPM level) of a transporter gene to decrease as the pathogen grew throughout the host. This is why we mentioned it.
• Why did you choose Phytophthora infestans for comparison? We chose to compare our data with Phy. infestans because there was a paper measuring transporter gene expression during infection on tomato plants. We were curious to find any similarities in the expression patterns of the transporter genes. Phy. infestans is an oomycete but not an obligate plant pathogen.
• In the section “Possible functions for the highly-expressed Peronospora transporter genes” the authors need to be careful when referring to a gene which function is characterized or is simply a approximation by homology. Please be clear in all your discussion. “RMX69262 encodes a putative 2-oxoglutarate/malate carrier protein, also known as the mitochondrial dicarboxylate-tricarboxylate carrier (DTC) protein (49). The RMX69262 protein from P. effusa shares 38% identity with A. thaliana DTC (At5g19760, BLASTP E value = 2e-67), which specifically transports protonated citrate and unprotonated malate (49); DTCs can also transport oxaloacetate, oxoglutarate, isocitrate cis-aconitate, and trans-aconitate (49).” This is a good example that you can replicate along all this part of the discussion. We have gone through this section of the manuscript to make sure that we state a putative or probable function. Verification of the possible function will need to be pursued in later studies.
• In the comparison to Phytophthora infestans, why are you comparing your set of “late” infection time point to 2 DPI? The necrotrophic phase begins in P. infestans at 3 dpi? (You do not have this info in the methods). In the paper by Abrahamian et al. 2016, the P. infestans should be at the biotrophic at 2 DPI in tomato leaves. This was stated in the results and discussion section, in the first paragraph below the subtitle 'Possible functions for the highly-expressed Peronospora transporter genes'.
• How the expression numbers in P. infestans study that you present in table 2 compare to your study? The expression value and ranking for each P. infestans gene is listed in the far right column of Table 3. It would be difficult to compare expression values from the P. infestans and our study directly, and this is why we utilized the ranking as a means of comparison. This was stated in the results and discussion section, in the first paragraph below the subtitle 'Possible functions for the highly-expressed Peronospora transporter genes'.
• Line 256: “The results of a previous study indicated that both ADP/ATP translocase and phosphate carrier protein levels were high in A. thaliana (43)…” In what condition?.. This sentence was modified to state that the ADP/ATP translocase and the phosphate carrier were the most abundant proteins in the inner mitochondrial membrane of Arabidopsis plants according to Fuchs et al. 2020. “suggesting that these proteins were part of a metabolic interaction (44).” What kind of metabolic interaction? The phrase about the metabolic interaction was removed, as Kunji et al. suggest that the ADP/ATP translocase has no functional interactions with any other proteins (https://doi.org/10.1016/j.bbamcr.2016.03.015).

Minor changes:
Line 102 and 117: Specify ~100.000 spores/ mL. Changed as suggested.
Line 228: Typo. Is 2 or 5 DPI. Changed to 5 DPI.

6. PLOS authors have the option to publish the peer review history of their article ([what does this mean?](https://gcc02.safelinks.protection.outlook.com/?url=https%3A%2F%2Fjournals.plos.org%2Fplosone%2Fs%2Feditorial-and-peer-review-process%23loc-peer-review-history&data=05%7C01%7C%7C148dee5cd8ca4bf0dc6908dad154138d%7Ced5b36e701ee4ebc867ee03cfa0d4697%7C0%7C0%7C638052456770993665%7CUnknown%7CTWFpbGZsb3d8eyJWIjoiMC4wLjAwMDAiLCJQIjoiV2luMzIiLCJBTiI6Ik1haWwiLCJXVCI6Mn0%3D%7C3000%7C%7C%7C&sdata=vXfcgfVa7jXNjl2hjjhMxXhiLL8cFEfDnSmtRfmLKbU%3D&reserved=0)). If published, this will include your full peer review and any attached files.


If you choose “no”, your identity will remain anonymous but your review may still be made public.


**Do you want your identity to be public for this peer review?** For information about this choice, including consent withdrawal, please see our [Privacy Policy](https://gcc02.safelinks.protection.outlook.com/?url=https%3A%2F%2Fwww.plos.org%2Fprivacy-policy&data=05%7C01%7C%7C148dee5cd8ca4bf0dc6908dad154138d%7Ced5b36e701ee4ebc867ee03cfa0d4697%7C0%7C0%7C638052456770993665%7CUnknown%7CTWFpbGZsb3d8eyJWIjoiMC4wLjAwMDAiLCJQIjoiV2luMzIiLCJBTiI6Ik1haWwiLCJXVCI6Mn0%3D%7C3000%7C%7C%7C&sdata=Okw7GD2q84BGxAtbIfCr2aE1a3gzy0859j7IwOJH6WI%3D&reserved=0).

Reviewer #1: No

Reviewer #2: No

[NOTE: If reviewer comments were submitted as an attachment file, they will be attached to this email and accessible via the submission site. Please log into your account, locate the manuscript record, and check for the action link "View Attachments". If this link does not appear, there are no attachment files.]

While revising your submission, please upload your figure files to the Preflight Analysis and Conversion Engine (PACE) digital diagnostic tool, [https://pacev2.apexcovantage.com/](https://gcc02.safelinks.protection.outlook.com/?url=https%3A%2F%2Fpacev2.apexcovantage.com%2F&data=05%7C01%7C%7C148dee5cd8ca4bf0dc6908dad154138d%7Ced5b36e701ee4ebc867ee03cfa0d4697%7C0%7C0%7C638052456771149881%7CUnknown%7CTWFpbGZsb3d8eyJWIjoiMC4wLjAwMDAiLCJQIjoiV2luMzIiLCJBTiI6Ik1haWwiLCJXVCI6Mn0%3D%7C3000%7C%7C%7C&sdata=9ognVuB7EqtOtk8o2bG5nRdj0a4Ypz1HMUIE287MiIo%3D&reserved=0). PACE helps ensure that figures meet PLOS requirements. To use PACE, you must first register as a user. Registration is free. Then, login and navigate to the UPLOAD tab, where you will find detailed instructions on how to use the tool. If you encounter any issues or have any questions when using PACE, please email PLOS at [figures@plos.org](mailto:figures@plos.org). Please note that Supporting Information files do not need this step.

*In compliance with data protection regulations, you may request that we remove your personal registration details at any time.* [*(Remove my information/details)*](https://gcc02.safelinks.protection.outlook.com/?url=https%3A%2F%2Fwww.editorialmanager.com%2Fpone%2Flogin.asp%3Fa%3Dr&data=05%7C01%7C%7C148dee5cd8ca4bf0dc6908dad154138d%7Ced5b36e701ee4ebc867ee03cfa0d4697%7C0%7C0%7C638052456771149881%7CUnknown%7CTWFpbGZsb3d8eyJWIjoiMC4wLjAwMDAiLCJQIjoiV2luMzIiLCJBTiI6Ik1haWwiLCJXVCI6Mn0%3D%7C3000%7C%7C%7C&sdata=1dPFqX%2FlJsbnKi%2FhLJP2xJMexrP02FAZhDcYM47mQUM%3D&reserved=0)*. Please contact the publication office if you have any questions.*
