# Supplementary material for: A comparison of transporter gene expression in three species of Peronospora plant pathogens during host infection
Source: PLoS One. 2023 Jun 1;18(6):e0285685. doi: 10.1371/journal.pone.0285685 (PMC10234565; doi:10.1371/journal.pone.0285685)
Supplement: Attachment — Submitted filename: Responses number two.docx [file pone.0285685.s012.docx]

PONE-D-22-25502R1

A comparison of transporter gene expression in three species of Peronospora plant pathogens during host infection

PLOS ONE

Dear Dr. Johnson,

Thank you for submitting your manuscript to PLOS ONE. After careful consideration, we feel that it has merit but does not fully meet PLOS ONE’s publication criteria as it currently stands. Therefore, we invite you to submit a revised version of the manuscript that addresses the points raised during the review process.

All responses by the authors are highlighted in red.

Please, improve the abstract, as reviewer #2 suggests, by removing of modifying the following two sentences "Several of the 16 highly-expressed transporter genes identified in this study were also highly-expressed in Phytophthora infestans during biotrophic infection of  tomato leaves as measured in a previously published study. Comparisons to other studied transporters indicated that most of the 16 highly-expressed transporter genes have a putative function that will need to be confirmed in future experiments." These sentences were removed from the abstract. Also, do not forget to clarify figure 1 to make it informative, as suggested by the reviewer #2. We removed the bar graphs from figure 1 as recommended by reviewer #2. Please, also carefully address the remaining remarks of the reviewer #1. We addressed these remarks below.

Other changes in the R2 version not pointed out by the reviewers:

Numbering is from the "clean" manuscript

In the abstract

27 highly expressed, not highly-expressed

In the last paragraph of section titled "Differential expression of *Peronospora* transporter genes during interactions between oomycete pathogens and their susceptible hosts"

269 each plant host or may be due to variations in metabolism in each pathogen.

Comma after host removed

Please submit your revised manuscript by Apr 14 2023 11:59PM. If you will need more time than this to complete your revisions, please reply to this message or contact the journal office at [plosone@plos.org](mailto:plosone@plos.org). When you're ready to submit your revision, log on to <https://www.editorialmanager.com/pone/> and select the 'Submissions Needing Revision' folder to locate your manuscript file.

Please include the following items when submitting your revised manuscript:

- A rebuttal letter that responds to each point raised by the academic editor and reviewer(s). You should upload this letter as a separate file labeled 'Response to Reviewers'.
- A marked-up copy of your manuscript that highlights changes made to the original version. You should upload this as a separate file labeled 'Revised Manuscript with Track Changes'.
- An unmarked version of your revised paper without tracked changes. You should upload this as a separate file labeled 'Manuscript'.

If you would like to make changes to your financial disclosure, please include your updated statement in your cover letter. Guidelines for resubmitting your figure files are available below the reviewer comments at the end of this letter.

If applicable, we recommend that you deposit your laboratory protocols in protocols.io to enhance the reproducibility of your results. Protocols.io assigns your protocol its own identifier (DOI) so that it can be cited independently in the future. For instructions see: [https://journals.plos.org/plosone/s/submission-guidelines#loc-laboratory-protocols](https://gcc02.safelinks.protection.outlook.com/?url=https%3A%2F%2Fjournals.plos.org%2Fplosone%2Fs%2Fsubmission-guidelines%23loc-laboratory-protocols&data=05%7C01%7C%7C7665ac05b3d04ba4188c08db19a01940%7Ced5b36e701ee4ebc867ee03cfa0d4697%7C0%7C0%7C638131947629139641%7CUnknown%7CTWFpbGZsb3d8eyJWIjoiMC4wLjAwMDAiLCJQIjoiV2luMzIiLCJBTiI6Ik1haWwiLCJXVCI6Mn0%3D%7C3000%7C%7C%7C&sdata=sZeJcgod5FkgPMFY%2BN7nMNvDWCEU7Ahd9ci20SItDEk%3D&reserved=0). Additionally, PLOS ONE offers an option for publishing peer-reviewed Lab Protocol articles, which describe protocols hosted on protocols.io. Read more information on sharing protocols at [https://plos.org/protocols?utm_medium=editorial-email&utm_source=authorletters&utm_campaign=protocols](https://gcc02.safelinks.protection.outlook.com/?url=https%3A%2F%2Fplos.org%2Fprotocols%3Futm_medium%3Deditorial-email%26utm_source%3Dauthorletters%26utm_campaign%3Dprotocols&data=05%7C01%7C%7C7665ac05b3d04ba4188c08db19a01940%7Ced5b36e701ee4ebc867ee03cfa0d4697%7C0%7C0%7C638131947629139641%7CUnknown%7CTWFpbGZsb3d8eyJWIjoiMC4wLjAwMDAiLCJQIjoiV2luMzIiLCJBTiI6Ik1haWwiLCJXVCI6Mn0%3D%7C3000%7C%7C%7C&sdata=Up%2BF%2FJyyhhHNhU9z0q%2BNwR%2BO7sk%2BB9EGHnPMldK1yeU%3D&reserved=0).

We look forward to receiving your revised manuscript.

Kind regards,

Hernâni Gerós, PhD
Academic Editor
PLOS ONE

Journal Requirements:

Please review your reference list to ensure that it is complete and correct. If you have cited papers that have been retracted, please include the rationale for doing so in the manuscript text, or remove these references and replace them with relevant current references. Any changes to the reference list should be mentioned in the rebuttal letter that accompanies your revised manuscript. If you need to cite a retracted article, indicate the article’s retracted status in the References list and also include a citation and full reference for the retraction notice.
We checked all the cited literature and none of these papers were retracted to our knowledge. The paper by Constantinescu could not be found in an electronic form. A colleague (Dr. Joanne Crouch) provided this article for us. It has been cited 97 times according to Google Scholar (as of 3-13-23). In a recent paper (Validation of five *Peronospora* species names; Mycotaxon, Volume 136(4), October-December 2021, pp. 785-788) Davis and Crouch write this about the paper written by Constantinescu: "Constantinescu (1991) provides the most comprehensive list of *Peronospora* names and is an invaluable resource to those studying the genus."
Not much information regarding the legitimacy of Raabe et al. 1952 could be found. Therefore, a modern article focused on spinach downy mildew disease was used in its place (Kandel et al. 2019 Plant Disease 103: 791-803).

[Note: HTML markup is below. Please do not edit.]

Reviewers' comments:

Reviewer's Responses to Questions

**Comments to the Author**

1. If the authors have adequately addressed your comments raised in a previous round of review and you feel that this manuscript is now acceptable for publication, you may indicate that here to bypass the “Comments to the Author” section, enter your conflict of interest statement in the “Confidential to Editor” section, and submit your "Accept" recommendation.

Reviewer #1: (No Response)

Reviewer #2: (No Response)

2. Is the manuscript technically sound, and do the data support the conclusions?

The manuscript must describe a technically sound piece of scientific research with data that supports the conclusions. Experiments must have been conducted rigorously, with appropriate controls, replication, and sample sizes. The conclusions must be drawn appropriately based on the data presented.

Reviewer #1: Partly

Reviewer #2: Yes

3. Has the statistical analysis been performed appropriately and rigorously?

Reviewer #1: Yes

Reviewer #2: Yes

4. Have the authors made all data underlying the findings in their manuscript fully available?

The [PLOS Data policy](https://gcc02.safelinks.protection.outlook.com/?url=http%3A%2F%2Fwww.plosone.org%2Fstatic%2Fpolicies.action%23sharing&data=05%7C01%7C%7C7665ac05b3d04ba4188c08db19a01940%7Ced5b36e701ee4ebc867ee03cfa0d4697%7C0%7C0%7C638131947629139641%7CUnknown%7CTWFpbGZsb3d8eyJWIjoiMC4wLjAwMDAiLCJQIjoiV2luMzIiLCJBTiI6Ik1haWwiLCJXVCI6Mn0%3D%7C3000%7C%7C%7C&sdata=4yDcIrDmGuXv0PifS4RDWaLO2RxIBQJObw9p0p6OWY8%3D&reserved=0) requires authors to make all data underlying the findings described in their manuscript fully available without restriction, with rare exception (please refer to the Data Availability Statement in the manuscript PDF file). The data should be provided as part of the manuscript or its supporting information, or deposited to a public repository. For example, in addition to summary statistics, the data points behind means, medians and variance measures should be available. If there are restrictions on publicly sharing data—e.g. participant privacy or use of data from a third party—those must be specified.

Reviewer #1: Yes

Reviewer #2: Yes

5. Is the manuscript presented in an intelligible fashion and written in standard English?

PLOS ONE does not copyedit accepted manuscripts, so the language in submitted articles must be clear, correct, and unambiguous. Any typographical or grammatical errors should be corrected at revision, so please note any specific errors here.

Reviewer #1: Yes

Reviewer #2: Yes

6. Review Comments to the Author

Please use the space provided to explain your answers to the questions above. You may also include additional comments for the author, including concerns about dual publication, research ethics, or publication ethics. (Please upload your review as an attachment if it exceeds 20,000 characters)

Reviewer #1: Dear Authors,
Your revised PlosOne manuscript on Peronospora transporter gene expression was extended for my peer-review. Thank you for your work on this submission. At this stage, I regret to point out that not all comments were addressed satisfactorily or in uniform manner, and new issues arose. All those are pointed out below, with bylines identifying their occurrence. I am disappointed to see many inconsistencies throughout the manuscript, which implies inattention to detail, which in turn is not reflecting well on the Authors and quality of their work.

Answer to Reviewers (Disease vs. Pathogen)
Still present in this version of the manuscript, although Authors responded that it was corrected throughout. l.52,505,506. All these instances were corrected. We also searched the entire document for occurrences of “downy mildew” to make sure that the usage was correct.

"while" in the meaning of "whereas" - still present despite Authors response of correcting throughout. We checked the entire manuscript and replaced “while” with “whereas” in all instances.

RNA isolation / quality checks not described briefly at all - just refers to a previous paper. Mentioning a kit name + manufacturer and whether their protocol was followed is preferred over referring to another (possibly paywalled) publication. That's against Open Science. A brief description of the RNA isolation procedure and subsequent quality checks were added to the manuscript.

Authors agreed that "significant" in the meaning of "substantial" or similar was not a good word choice, yet still present in R1. “Significant” has been replaced with “substantial” in all locations in the manuscript.

81 Further insights can add to context here: DOI 10.1016/bs.adgen.2020.03.001; 10.1101/2020.02.12.941195 We added two sentences that discussed some relative points about metabolic networks of obligate plant pathogens from the preprint manuscript by Rodenburg et al. 2020 (10.1101/2020.02.12.941195). The book chapter by McGowan and Fitzpatrick (2020, 10.1016/bs.adgen.2020.03.001) did not talk much about metabolic networks, so no text was added to the manuscript here.

116 leaves( )with. This typo was fixed.

104 vs 119 unit inexistency. The unit mL was changed to ml.

107 (170) of( )-5. This typo was fixed.

179 several (add: possible; conceivable; or similar). “Several” was replaced with “similar”.

188 the most (Consider: the highest number of). The change was made as recommended.

192 Inconsistent use of serial comma (a.k.a., Oxford comma). A comma was added after “symporter”.

206 the top 50 (Here and in similar places; Correct throughout for the full meaning). More description was added here and in all locations, including Figure 2.

209;228;264 Most (Majority). “Most” was replaced with “The class of transporters that was most frequently found in the top 50 most highly expressed genes” for the *P* *effusa* data. A similar phrase was added to describe the most frequently found transporter class for the *P belbahrii* and *P* *tabacina* transporter gene analyses. Majority substituted for most at line 264.

212,213 tubulin B; dehydrogenase alpha etc (Absent from Table 1. Or inconsistently named). Corrections were made for these two gene names in the text as well as in Table 2.

214 [MAJOR] The title should indicate what is shown in this table. What do all these numbers represent and how were they generated? Do not make the reader look for explanations, make this a reader-friendly manuscript. The title of Table 2 was modified. An explanation about the gene names was added to the Table 2 legend.

221 The (expression rates of). This sentence was changed as recommended.

222 Inconsistent double spacing. This double spacing was corrected.

235;411 significant (see Response to Reviewers comment). “Significant” was replaced with “substantial” at these two locations.

240 5 for 5 the DPI (what is meant here?). The order of the words was changed here.

247 Some (What criteria were applied to select those "some"?). The criteria were described in the modified sentence.

251 An exemplary way to define and use throughout for the top X genes. Noted.

252 timepoints (were "stages of infection" or similar meant here and in 254?). Timepoints was rephrased in both instances.

254 [MAJOR] 7 is most definitely NOT the most of 16. Rephrase for precision. The following edit was done: “in common, seven were MCs, as shown in”.

262-267 [MAJOR] Is this passage referring to [47] or to the results of this very study? Rephrase for clarity. The Rodenberg et al. paper [47] was cited several times as needed in this revised paragraph.

264 91 pathways (data not shown or [47]?). The 91 pathways were from [47]. The sentence was modified accordingly.

292 Abrahamian - inconsistent citation style. The data of Abrahamian et al. was not cited at this location because Reviewer #2 suggested to remove all the *Phytophthora* *infestans* data from Table 3.

451-452 3-fold higher compared to (correct the grammar please; was " than the" meant here?). The grammar was corrected here, and some additional text was added for clarity.

480,505,506 "downy mildew" in the meaning of the causal pathogen(s). For each occurrence, “pathogen” was added after “downy mildew”.

496 they plays role (correct the grammar please). In this sentence, “they” was replaced with “each transporter”.

504 pathogens . (spurious space). This extra space was removed.

References - inconsistencies in journal names; pathogen italics, etc. The references were formatted for the standard PLoS One style using EndNote software.

Reviewer #2: Reviewer: Dear editor and authors,

The authors developed in this study a comparison of gene expression of transporter proteins of three different Peronospora species during infection. The manuscript is simple, clear, well structured and written. For the study, they performed the infection experiments for one species and used two different public databases of published experiments. Therefore, this study is partially based on original scientific research but the analysis of the public databases is slightly interesting. The standard of the experiments is of high degree and the methods are sufficiently detailed, as well as the statistical analysis. The conclusions are based in the results and are well presented.

In the next line I present a series of recommendations and questions to debate with the authors.

• Line 26: This type of discussion is not adequate for the abstract. This specific line is a summary of our data in two different sentences. These data are mentioned in more detail in the discussion section.

RESPONSE: Line 29 “Several of the 16 highly-expressed transporter…will need to be confirmed in future experiments.” The sentences are these. This is a discussion sentence that do not add relevant information for the abstract, specially the second one which is innocuous for the discussion. Please remove the sentences. You can wrap it up just with the last sentence. We removed the two sentences from the abstract.

• What is the rational of study these 3 pathogen species? Downy mildew disease causes major losses on economically important plants, such as those in this study; it is important to find out more detail about how these pathogens infect their hosts. In addition, we thought it would be of interest to analyze transporter gene expression in these three pathosystems to see if there are similarities or differences in the most highly expressed transporter genes during host infection.

RESPONSE: So, the authors just selected downy mildew to study and then randomly selected 3 species affected by it. Our government established research project was focused on downy mildew disease at the time. That is why we chose to study downy mildew. The three species were chosen because transcriptome data of two species were available for analysis. The corresponding author published research on the basil downy mildew pathogen transcriptome in 2022. Transcriptome data for the spinach downy mildew pathogen was in NCBI GenBank. The current study generated transcriptome data for the tobacco downy mildew pathogen.

• Can the authors explain the discrepancies regarding the days after inoculation of the samples? The studies of the basil, spinach and tobacco were independently completed. However, we felt that we could analyze this data together in this paper since the early timepoint (2 or 3 DPI) or the late timepoint (5, 6, or 7 DPI) only deviated one day or less than one day.

RESPONSE: I am not completely comfortable with this discrepancy, however is plausible that the physiological stage of the pathogen is not affected in such a manner that flaw this comparison. Noted.

• I cannot understand the value of the bars graphs presented in figure 1 without the names of the genes. We think it would be difficult to include the names of 50 genes on the horizontal axis. Readers would be able to find the gene name using the supplementary tables S3-S5.

RESPONSE: If you do not have the name of the genes what is the real purpose of the graphic? To have “one more result”? If is not informative it should not be included. At least use your nomenclature (the numbers, 1, 2, etc) to identify the genes in the respective supplementary table. The bar graphs were removed from Figure 1, and the figure was reformatted.

• The gene code presented in table 1 is the “same” for homologous genes? Is confusing the nomenclature in the text differs from the table. Maybe you can opt to introduce the specie gene name and its homologue. Admittedly it was difficult to identify a system for naming homologous genes. We settled on using the P. effusa RMX or RQM number as the homologous gene identifier in each Peronospora species. Readers can notice that the RMX/RQM naming system is used in Tables 2-4 for cross comparison. We think that we went through the Tables sequentially in the manuscript and introduce protein designations in the text (which were species specific) as needed. Moreover, Reviewer #1 did not mention a problem with our homologous gene identifiers.

RESPONSE: Is not a problem, is just confusing, is difficult to keep up. Noted. We also edited some of the manuscript text where it referred to RMX or RQM genes for clarity. RMX or RQM gene names are used throughout the manuscript until the section “In depth transporter protein analysis”.

• Line 215: “The TPM level of the MC gene PBEL_00715, as well as that of the actin gene, decreased from 3 to 6 DPI.” Why is this line relevant? Are these the only genes that decrease from 3 to 6 dpi? We thought it was interesting to find the expression (TPM level) of a transporter gene to decrease as the pathogen grew throughout the host. This is why we mentioned it.

RESPONSE: But it was the only gene that reduced the expression or the only transporter? This sentence was deleted, as it is not that necessary for the whole story.

• Why did you choose Phytophthora infestans for comparison? We chose to compare our data with Phy. infestans because there was a paper measuring transporter gene expression during infection on tomato plants. We were curious to find any similarities in the expression patterns of the transporter genes. Phy. infestans is an oomycete but not an obligate plant pathogen.

RESPONSE: But Phytophthora infestans is a hemibiotrophic pathogen. Even just using the biotrophic phase is not completely suitable. The entire column with P. infestans data, as well as the explanatory text, was removed from the table and manuscript, respectively.


The authors just need to adjust a few things to complete the publication. Thank you for your assistance in making our manuscript better.

7. PLOS authors have the option to publish the peer review history of their article ([what does this mean?](https://gcc02.safelinks.protection.outlook.com/?url=https%3A%2F%2Fjournals.plos.org%2Fplosone%2Fs%2Feditorial-and-peer-review-process%23loc-peer-review-history&data=05%7C01%7C%7C7665ac05b3d04ba4188c08db19a01940%7Ced5b36e701ee4ebc867ee03cfa0d4697%7C0%7C0%7C638131947629139641%7CUnknown%7CTWFpbGZsb3d8eyJWIjoiMC4wLjAwMDAiLCJQIjoiV2luMzIiLCJBTiI6Ik1haWwiLCJXVCI6Mn0%3D%7C3000%7C%7C%7C&sdata=9lDahvcIGqRo7%2BKlrnmwTe8LGOqY5J52OKS4V0hb9DE%3D&reserved=0)). If published, this will include your full peer review and any attached files.


If you choose “no”, your identity will remain anonymous but your review may still be made public.


**Do you want your identity to be public for this peer review?** For information about this choice, including consent withdrawal, please see our [Privacy Policy](https://gcc02.safelinks.protection.outlook.com/?url=https%3A%2F%2Fwww.plos.org%2Fprivacy-policy&data=05%7C01%7C%7C7665ac05b3d04ba4188c08db19a01940%7Ced5b36e701ee4ebc867ee03cfa0d4697%7C0%7C0%7C638131947629139641%7CUnknown%7CTWFpbGZsb3d8eyJWIjoiMC4wLjAwMDAiLCJQIjoiV2luMzIiLCJBTiI6Ik1haWwiLCJXVCI6Mn0%3D%7C3000%7C%7C%7C&sdata=P2G9gM9JhZO%2F8PSFzI5D%2FkxdWY%2FnjMzaKGjvv8RTwk4%3D&reserved=0).

Reviewer #1: **Yes:** Marcin Nowicki

Reviewer #2: No

[NOTE: If reviewer comments were submitted as an attachment file, they will be attached to this email and accessible via the submission site. Please log into your account, locate the manuscript record, and check for the action link "View Attachments". If this link does not appear, there are no attachment files.]

While revising your submission, please upload your figure files to the Preflight Analysis and Conversion Engine (PACE) digital diagnostic tool, [https://pacev2.apexcovantage.com/](https://gcc02.safelinks.protection.outlook.com/?url=https%3A%2F%2Fpacev2.apexcovantage.com%2F&data=05%7C01%7C%7C7665ac05b3d04ba4188c08db19a01940%7Ced5b36e701ee4ebc867ee03cfa0d4697%7C0%7C0%7C638131947629295869%7CUnknown%7CTWFpbGZsb3d8eyJWIjoiMC4wLjAwMDAiLCJQIjoiV2luMzIiLCJBTiI6Ik1haWwiLCJXVCI6Mn0%3D%7C3000%7C%7C%7C&sdata=yTXpi4m%2FPuiguI2h5Cp1yWFMRR4zuWmNqcn27t3fi84%3D&reserved=0). PACE helps ensure that figures meet PLOS requirements. To use PACE, you must first register as a user. Registration is free. Then, login and navigate to the UPLOAD tab, where you will find detailed instructions on how to use the tool. If you encounter any issues or have any questions when using PACE, please email PLOS at [figures@plos.org](mailto:figures@plos.org). Please note that Supporting Information files do not need this step.

*In compliance with data protection regulations, you may request that we remove your personal registration details at any time.* [*(Remove my information/details)*](https://gcc02.safelinks.protection.outlook.com/?url=https%3A%2F%2Fwww.editorialmanager.com%2Fpone%2Flogin.asp%3Fa%3Dr&data=05%7C01%7C%7C7665ac05b3d04ba4188c08db19a01940%7Ced5b36e701ee4ebc867ee03cfa0d4697%7C0%7C0%7C638131947629295869%7CUnknown%7CTWFpbGZsb3d8eyJWIjoiMC4wLjAwMDAiLCJQIjoiV2luMzIiLCJBTiI6Ik1haWwiLCJXVCI6Mn0%3D%7C3000%7C%7C%7C&sdata=M%2B5Wxo18doMLsD%2B%2FkEsiVbgQMz3Z810QuKdoU6bPDVs%3D&reserved=0)*. Please contact the publication office if you have any questions.*
